# Contrasting area and yield responses to extreme climate contributes to climate-resilient rice production in Asia

**DOI:** 10.1038/s41598-023-33413-7

**Published:** 2023-04-17

**Authors:** Nanae Hosokawa, Yasuhiro Doi, Wonsik Kim, Toshichika Iizumi

**Affiliations:** 1 Institute for Agro-Environmental Sciences, National Agriculture and Food Research Organization, Tsukuba, Ibaraki 305-8604 Japan; 2 Forestry and Forest Products Research Institute, Forest Research and Management Organization, Tsukuba, Ibaraki 305-8687 Japan; 3 CSJ Co. Ltd., Shibuya-Ku, Tokyo, 151-0053 Japan

**Keywords:** Climate sciences, Environmental sciences

## Abstract

Climate impacts on crop production components other than yield, i.e., area and cropping intensity, remain under-studied. Here, we clarify climate-crop area relationships by analyzing subnational census area and yield data for six multi-rice cropping countries in South and Southeast Asia. Extreme climate has a greater influence on the departure of area and yield from long-term trends than the average seasonal climate; precipitation and temperature in the sowing period of the wet/rainfed season have a greater influence on variability of the total annual area than in the growing period. In 57% of the country-scenario cases showing significant changes in area and/or yield, the directions of the area and yield responses to climate are not synchronized, deriving non-significant production changes under projected climates. Climate-area relationships not only limit production shocks, but also clarify uncertainties associated with climate mitigation of agricultural land, where area markedly affects the scale of mitigation.

## Introduction

Extreme weather and climate events often reduce crop production and trigger food shocks through interconnected global supply chains, exacerbating food security and nutrition in vulnerable regions of the world^1–3^. Numerous studies have focused on the impacts of climate on yields (production volume per unit harvested area and season) due to the primary contributions of yield on production increases in recent decades. However, as mentioned in previous studies^4–6^, an under-studied question remains: How does climate influence the production components other than yield—crop area (planted or harvested) and cropping intensity (number of harvests per year)?

Both crop area and cropping intensity are crucial for determining the climate impacts on production volume, particularly in multi-cropping^5–9^ and drought-prone regions^10^. To better capture production variability, understanding the impacts of climate on individual production components is important. Better understanding of these impacts leads ultimately to interventions and policies that are focused on moderating negative food price cascades and on increased food insecurity in vulnerable regions and among consumer groups^3,11,12^. It is possible to say that understanding of these impacts is important for land-based climate mitigation related to agriculture, because a decrease in the harvested area due to extreme climate events, relative to the implementation (planted) area, has not been considered. Indeed, even in recent land-use change modeling studies (ex.^13^ and the latest assessments of mitigation potentials for bioenergy with carbon capture and storage (BECCS, ex.^14^, the extent of the implementation area is a crucial factor affecting the scale of the mitigation effect.

**Figure 1:**
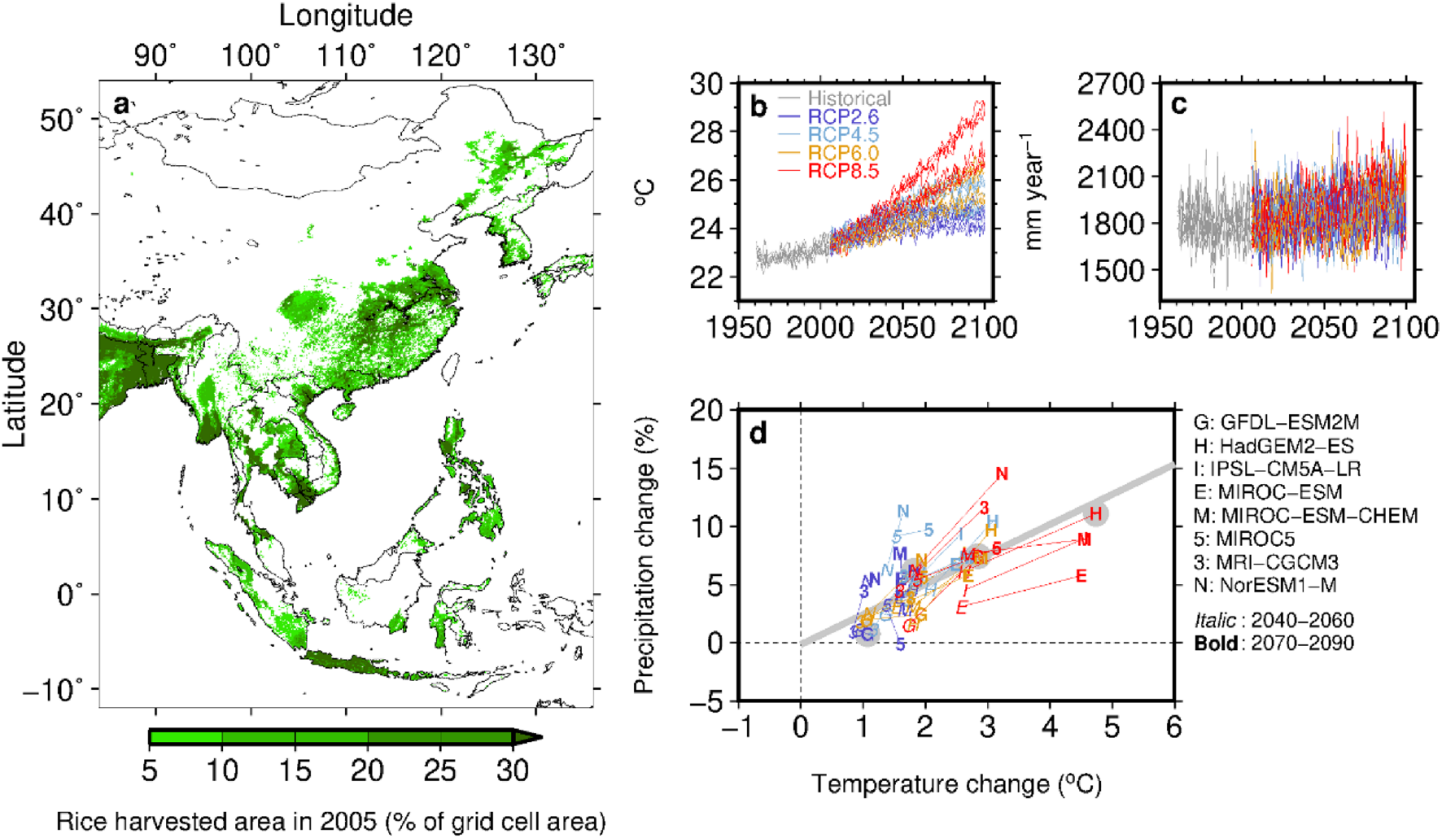
(**a**) The geographic distribution of rice area in 2005. (**b**,**c**) The projected annual mean temperature and total precipitation over the rice area in Asia (10° S–52° N; 85–135° E) from eight general circulation models (GCMs) under four representative concentration pathways (RCPs). (**d**) The relationship between the projected temperature and precipitation changes for the middle (2041–2060) and end (2081–2100) of the twenty-first century, relative to the period 1986–2005. The gray bold line is the best-fit line. Solid gray circles indicate the four climate scenarios selected for this study, consisting of two RCPs (RCP2.6 and RCP8.5) and two GCMs (GFDL-ESM2M and HadGEM2-ES). The map presented here was created from Generic Mapping Tools (GMT) version 4.5.18 (https://www.generic-mapping-tools.org/).

Here we attempt to clarify climate-area relationships and show that seasonal climate influences the characteristics of rice areas, as much as yields. The direction of the response to change in climate is often not synchronized between area and yield, and extreme climate have a greater effect on the departures of area and yield from long-term trends, than seasonal average climate. Indeed, multi-rice cropping regions account for one-third of the global rice area^15^. We studied six multi-rice cropping countries in South and Southeast Asia (Bangladesh, Indonesia, Malaysia, Myanmar, Philippines, and Thailand; Supplementary Table 1) that together accounting for a substantial portion of global rice production (25%) and area under rice cultivation (27%). The elastic net-regularized regression models associated annual area and yield anomalies with extreme and average precipitation and temperature indices for sowing and growing periods of wet and dry seasons (Supplementary Fig. 1). Climate projections that exhibit relatively large and small changes in long-term precipitation and temperature were selected (Fig. 1) and were used to characterize the area and yield responses to climate change and their relative contributions to production impacts.

### Area, yield, and production variabilities

**Figure 2:**
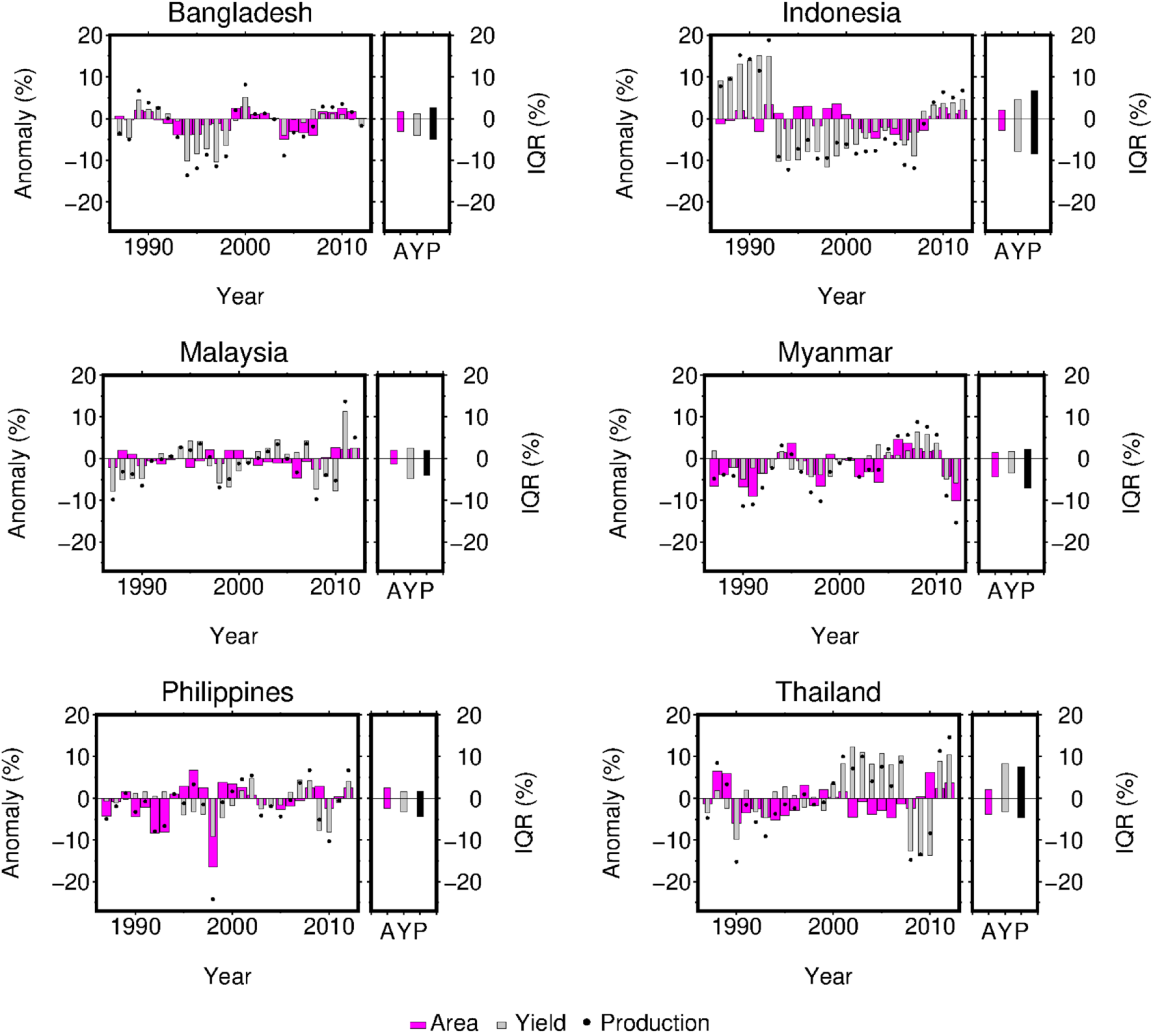
Reported anomalies of rice area, yield, and production. Inter-quantile ranges (IQR) are presented for area (A), yield (Y), and production (P).

The inter-quantile range (IQR) calculated from the census data for the period 1987–2012 revealed that the variability in crop area is not negligible, even when compared to yield variability. In Bangladesh, Myanmar, and the Philippines, the area variability values (IQL 4.8–5.8%) were comparable to the yield variability (4.7–5.2%), whereas the yield variability (7.4–12.5%) was 2.0-to-2.5-times larger than the area variability (3.3–5.9%) in the remaining three countries (Fig. 2, Supplementary Table 2). The production variability (6.0–15.1%) was larger than either the area or yield variability in all of the countries considered here, except for Malaysia (6.0%). Taken together, the results showed that the signs of the area and yield changed in the same direction in some years, but not always. Indeed, the area and yield anomalies were not significantly correlated with each other in four countries, with Bangladesh (R = 0.530; p = 0.005) and Myanmar (R = 0.480; p = 0.013) being exceptions (Supplementary Table 2).

### Model performance

Comparisons between the census data for the period 1987–2012 and out-of-sample reproduction using the elastic net regression models with actual weather conditions as the input showed that the model performance was satisfactorily high for both area and yield. For area, the mean R value between the countries was 0.990, with the minimum-to-maximum (min–max) range being from 0.970 for Malaysia to 0.997 for Myanmar (Supplementary Fig. 2). For yield, the corresponding mean value was 0.994 (0.989 for Thailand to 0.999 for Indonesia). These correlations were all significant at the 0.1% level. Root-mean-square errors (RMSE) ranged from 0.6 to 0.7% for area (mean 0.65%), and from 0.5 to 2.0% for yield (mean 0.9%), with the minimums and maximums corresponding to Thailand and the Philippines, respectively. Such high performance, even for the out-of-sample validation, is typically found in earlier studies that employed elastic net regression models (see “Methods”).

### Influence of climate on area and yield

**Figure 3:**
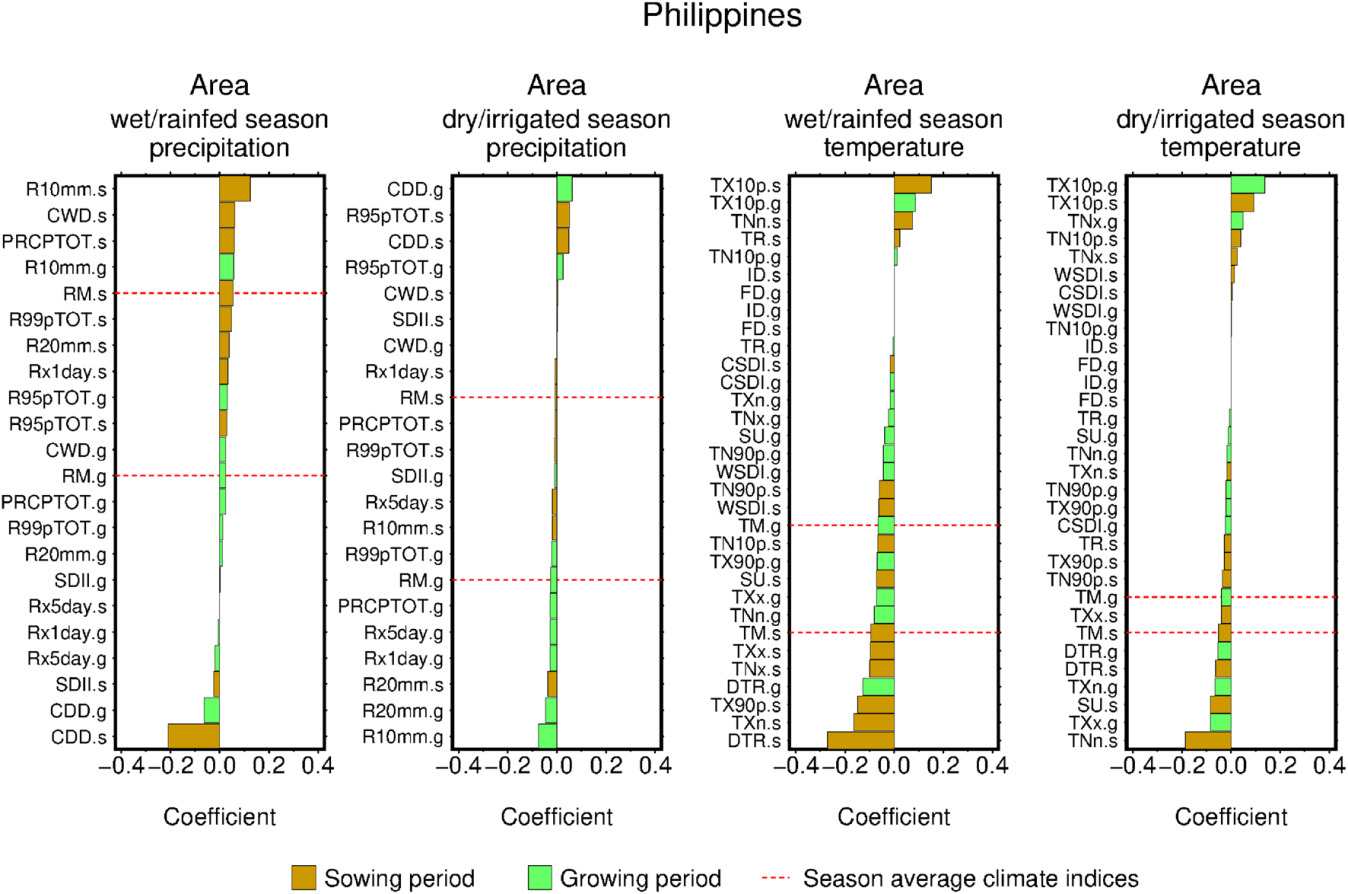
Area response to climate in the Philippines. The values indicate the standardized average regression coefficients of the elastic net regression models. The predictors with and without red dotted lines denote average and extreme climate indices, respectively. Brown indicates the sowing period; Green indicates the average of early and late growing period groups. The four panels show the different seasons (wet/rainfed and dry/irrigated) and climate variables (precipitation and temperature).

The estimated regression coefficients clarified the effect of climate on the current area and yield. If we use the Philippines as a representative example, then the following two features are apparent. First, the average climate indices are rarely ranked as being among the top factors affecting area and yield, either negatively or positively (three climate indices for each sign; the bars with and without red dotted lines in Fig. 3 and Supplementary Fig. S3). These findings indicate that extreme climate, regardless of that in wet and dry seasons, has a larger influence on area than the average seasonal climate. Second, compared to the growing period of the wet season, many more precipitation and temperature indices were identified as factors that most affected area, either positively or negatively, during the sowing period of the wet season (the brown bars account for more than half of the top contributors in Fig. 3). These findings show the influence of climate during the sowing period of the wet/rainfed season on year-to-year variability in annual total crop area; this is considered to be reasonable since the reported extent of the wet-season area in the Philippines in 2020 was approximately 1.3-times larger than that of the dry-season area.

The features described above were relatively common among the studied countries. Extreme climate conditions had a marked effect on area in four out of six countries. Average seasonal precipitation ranked higher only in Indonesia and Thailand (Supplementary Fig. 4, Supplementary Table 3). A similar pattern was observed for yields in the four countries. However, the average seasonal precipitation ranked as the top contributor of yields in Bangladesh and Indonesia. Among other extreme climate indices, the number of consecutive dry days (CDD) was often the top factor that negatively affected area (Supplementary Fig. 5). In addition, the contributions from extremely wet days (R99pTOT), very wet days (R95pTOT), consecutive wet days (CWD), and heavy precipitation days (R10mm) were also frequently ranked highly, but the sign of the area response was mixed. While the area response towards the extreme temperature indices was also mixed, the coldest days (TXn) and cool days (TX10p) tended to increase area. For yield, CWD and CDD were frequently identified as having decreasing and increasing effects, respectively. TX10p and hottest days (TXx) were also frequently detected, but the signs of the yield response were mixed.

The influence of precipitation and temperature in the sowing period of the wet season on area was a common feature across the four countries, except for Malaysia and Myanmar (Supplementary Fig. 4, Supplementary Table 3). Both precipitation and temperature had an influence, either positive or negative, on area in the sowing period of the wet season than in the growing period of the same season (Supplementary Fig. 5). This influence of the climate on area in the sowing period was seldomly observed in the dry season. This is considered to be reasonable since planted area in dry season is largely determined by the availability of irrigation facilities and water.

### Projected contributions of area and yield to production

We projected climate change impacts on area and yield, as well as the resulting changes in production under four climate scenarios, with measures of the reliability of projection. The scenarios consisted of two Representative Concentration Pathways [RCP2.6 (r26) and RCP8 (r85)] and two general circulation models (GCMs) [GFDL-ESM2M (gG) and HadGEM2-ES (gH)]. r26gG and r85gH represent the scenarios with smaller and larger changes in temperature and precipitation, respectively. The remaining scenarios, i.e., r85gG and r26gH, fall into the middle of the two scenarios in terms of the amplitudes of the projected changes in temperature and precipitation (Fig. 1d).

**Figure 4:**
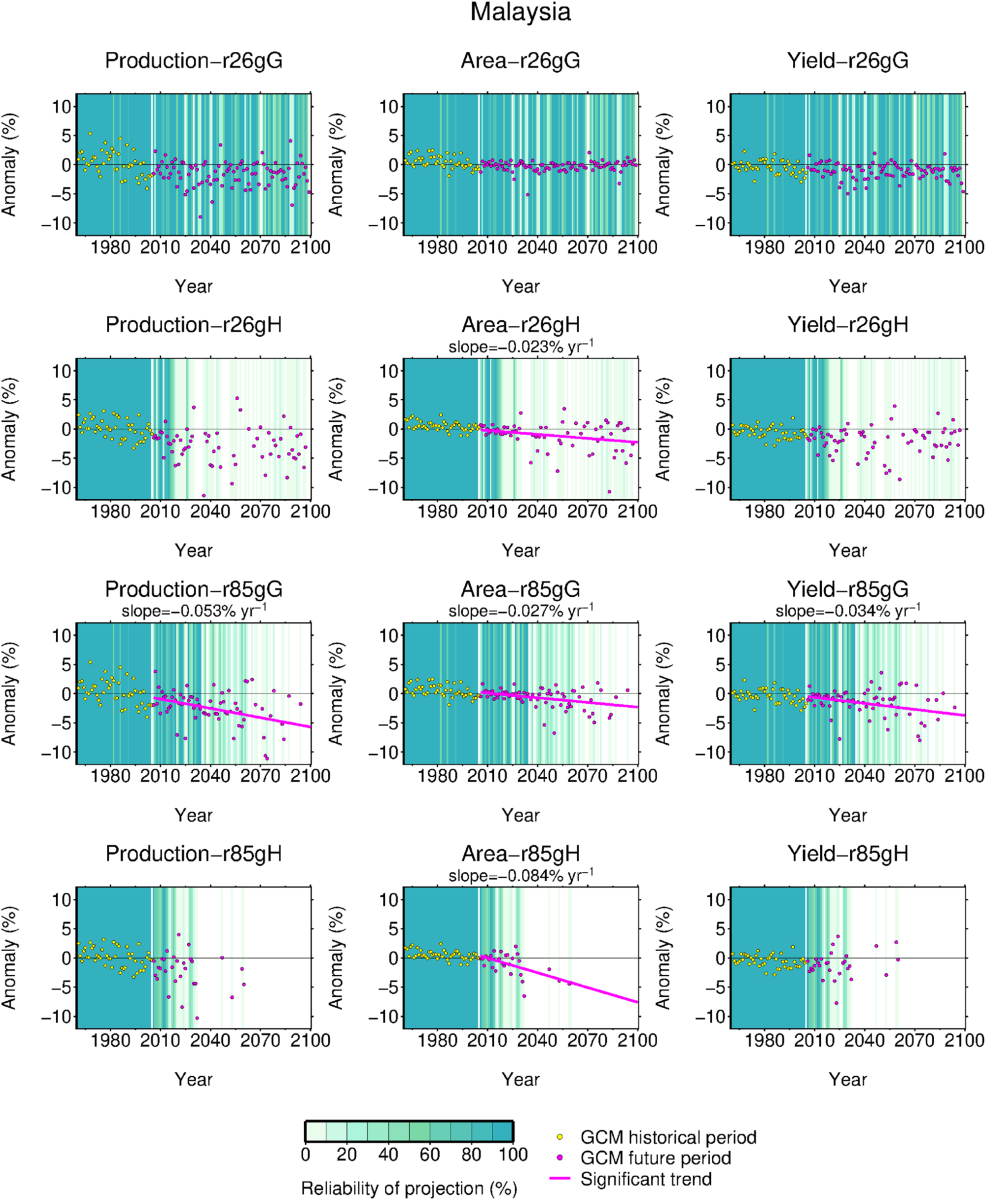
Projected changes in production, area, and yield for Malaysia. The heat map overlayed on each panel indicates the reliability of the projection. Lighter green and white columns indicate that many climatic predictors used to derive the projection fall outside the observed ranges; therefore, these projections are less reliable than projections in the darker green column. Less reliable projection samples are eliminated when fitting a trend line and testing the significance of the slope. Trend lines and the corresponding slope values are displayed only when a significant slope is obtained.

If Malaysia was taken as the representative example, then production was projected not to change significantly under three scenarios (r26gG, r26gH, and r85gH), of which two cases, namely changes in the projected area and yield, were not synchronized and did not produce significant production changes. Due to decreases in both area and yield, a production decrease was projected only under r85gG. The reliability of the projection was higher for r26gG than the remaining three scenarios. Using the elastic net regression models, we estimated the ratio of climatic predictors that fall within the observed min–max ranges to give readers a sense of the reliability of projection (see the heatmap overlayed on each panel of Fig. 4). In the heatmaps, the lighter the green color, the more the climatic predictors that were used to derive that projection fell outside the ranges of the training data, indicating that the projection was less reliable. As expected, the reliability of projection was high for the near future and for the scenario showing smaller changes in precipitation and temperature (r26gG).

Among the 24 cases consisting of six countries and four scenarios examined here, the projected production changes were not significant in most cases (21), mainly due to the low reliability of projection and resulting fewer samples in the statistical test. However, 7 out of 24 cases showed that either, or both, area and yield exhibited significant changes (Supplementary Figs. 6, 7); the rate of change per decade ranged from − 0.84 to + 1.11% for area and from − 0.34% to + 0.77% for yield (Fig. 4, Supplementary Fig. 6). Interestingly, 4 out of 7 cases, or 57%, showed non-significant production changes as a result of the non-synchronized area and yield responses to the projected changes in climate. These results show that production changes are not necessarily proportional to yield changes, and that climate-area relationships need to be considered to capture the climate impacts on production.

## Discussion

Comparisons with the literature indicate that the area and the yield responses to climate identified in this study are plausible, although the available studies are limited. The positive impacts of CWD and the negative impacts of CDD, both in sowing period of wet season, are apparent in Indonesia (Supplementary Fig. 4, Supplementary Table 3). These findings are consistent with those of Naylor et al.^7^ who reported that the area of wet-season rice for the last decades has increased in Indonesia in response to wetter-than-normal conditions. Koide et al.^8^ reported that, in the Philippines, wet-season rice area is positively correlated with pre-season precipitation, these findings are corroborated by those of this study, which showed that R10mm, CWD, and total wet day precipitation (PRECIPTOT) in the sowing period of the wet season had the highest effect on rice area (Fig. 3). For yield, extreme climate was reported to have a larger influence than seasonal average climate^16^, which is consistent with the results of this study. Finally, the projected yield changes in this study are considered to be reasonable given that these projections are distributed within the spread of the projections derived from process-based crop models (Supplementary Text). Due to the lack of previous studies on climate-area relationships, no comparisons are possible for the projected change in rice area.

Using multi-rice cropping countries in South and Southeast Asia as a case study, the findings of this study show that extreme weather and climate can cause departures from long-term trends in rice area. Although we studied only rice, the findings of this study have relevance to multi-cropping regions of the world, which account for 13% and 10% of the global wheat and maize areas, respectively^15^. Nevertheless, the question of ‘how does climate affect area?’ needs to be tackled in more process-resolvable ways in order to, ultimately, develop interventions and policies for safeguarding vulnerable regions and groups of producers. Our models suggest the existence of at least two impact pathways. One is that the extent of the area planted in the wet season is influenced by precipitation during the sowing period, which is likely related to water availability in rainfed conditions. The other is that the planted area decreases to the extent of the area at harvesting, irrespective of wet and dry seasons, due to damage caused by extreme climate events related to precipitation in the growing period, such as meteorological droughts and floods. Field workability and planting methods might also influence the former pathway. Agronomic practices, such as replanting and seed availability, may influence the latter pathway.

Despite a lack of studies, there is some supporting evidence for the abovementioned ideas. Related to the former pathway, the progress of rice transplantation in rainfed lowlands of Northeast Thailand is influenced by the precipitation accumulation rate after the start of the monsoons; therefore, delayed planting occurs in drier-than-normal years and decreases the area planted in the wet season. Producers therefore wait for precipitation to accumulate with sufficient time remaining for them to complete their rice harvest by the beginning of subsequent dry season when they change their planting method from transplanting to direct seeding; although this lowers yields, it is more time- and labor-saving than transplanting^17^. Related to the latter pathway, the area under cereal cultivation in Asia decreases when drought occurs^1^. For field crops in Iran, production is often reduced due to droughts through decrease in area harvested^10^. Such reductions in harvested areas are well-documented in the literature focusing on disaster damage and loss assessment in agriculture^18^. Compared to the influence of precipitation on an area, almost no information is currently available on how extreme and average temperatures influence crop area. Cohn et al.^5^ reported that higher temperatures decrease the crop area of maize-soybean in Brazil, however, relevant processes were not discussed.

The findings of this study are relevant not only to adaptation, but also to mitigation. While future precipitation projections are uncertain in rice-producing regions in Asia, 14% and 31% of the global cropland area are expected to experience drier and wetter climates by 2040, respectively^19^. These findings emphasize the need for adaptations to limit production reductions by moderating the impacts of climate change on area, which is rarely considered^20^. For climate mitigation, the extent of cropland area under a specific land-based mitigation measure is crucial to determine the scale of climate mitigation outcomes, this is true whether the measure is carbon sequestration into cropland soils or reduction of methane emissions from paddies^21–24^. Climate-induced area reductions could compromise outcomes from these mitigation measures at seasonal and longer time scales. For rice, the variability in the crop area associated with seasonal changes in climate could be on the order of 10% or more of the long-term trend, as shown in this study, which is considered to be non-negligible. The impacts of mean climate change on area are much less than the value reported here by two orders of magnitude (− 0.85% to + 0.06% per decade), although the impacts of large precipitation and temperature changes on area are not sufficiently considered in this study due to the lack of reliable projections. Therefore, future research is necessary to compare the scale of the effect of the area response to the changes in climate with other factors when assessing the outcomes of agricultural land-based mitigation measures.

## Methods

### Rice area and yield data

National and subnational census data on rice area harvested and yield in the six Asian countries for the period 1961–2012 were used (Supplementary Table 1). Data were collected from agricultural statistical yearbooks compiled by governmental agencies of the individual countries (see Refs.^25,26^ for the data sources). Since administrative units occasionally changed with time, we used those in 2010 for the analysis.

We detrended the annual time series of area and yield to remove the influences of changes in demand for rice, land-use changes, changes in input and output prices, and technological improvements, and focused primarily on the effects of climatic factors. The detrending was conducted separately for area and yield to allow different trend patterns (Supplementary Fig. 8). Such a case could occur, for instance, due to a decrease in area induced by urbanization and an increase in yield driven by intensification. We calculated percentage anomalies, relative to long-term trends, so that we could compare climate influences on area and yield and their relative contributions to production impacts. The use of percentage anomalies also enabled comparisons of climate contributions among the countries in which average area and yield levels differed in absolute terms.

For detrending, we adopted the double-filter approach, which involved first conducting the moving average with a 7-year window (*t − *3 to *t* + 3) and then applying locally weighted scatter-plot smoothing (LOWESS^27^ with a smoothing span (*f*) of 0.5 implemented in the R statistical package^28^. The double-filter approach was used because a visual check suggested that performing moving averaging alone or LOWESS alone was sometimes insufficient when the given time series was highly nonlinear.

### Extreme and average precipitation and temperature indices

We calculated 10 extreme precipitation indices and 15 extreme temperature indices defined by the Expert Team on Climate Change Detection and Indices (ETCCDI^29^ as well as seasonal average precipitation and temperature (Supplementary Table 4). These climate indices were computed for each period and season. We distinguished between multi-rice seasons. Double rice cropping, i.e., wet/rainfed season and dry/irrigated season, is performed in five countries. Triple rice cropping is performed in Bangladesh, and two seasons (Aman and Aus) were classified as the wet/rainfed season (Supplementary Table 1). Each season was divided into the sowing period and the growing period. As for the growing period, we further divided it into early and late groups. In total, we defined three periods per season (Supplementary Fig. 1): (1) *the sowing period*, from one month before the first month of the planting window to the last month of the planting window; (2) *the early growing period group*, from the first month of the planting window to the first month of the harvesting window; and (3) *the late growing period group*, from the last month of the planting window to the last month of the harvesting window. Although the sowing period and the early growing period group almost overlap, the presence of extreme climate events in the month prior to planting is the main difference between the two periods. Pre-planting climate conditions are unlikely to affect yields, but climate conditions just before and during planting influence water availability, field workability, and delay or accelerate the progress of planting and eventually the area planted^17,30,31^. We mainly used rice calendars from two sources: Agricultural Market Information System (AMIS) and the Global Information and Early Warning System (GIEWS) (Supplementary Table 1). In the case where the information for a country of interest is available for both sources, we used the AMIS calendar.

When calculating the climate indices for the projected climate, we kept the rice calendars the same as current conditions. Rice calendars may change along with climate change and adaptation. However, errors associated with this assumption were considered small when the calendars were used on a monthly basis, given that the observed shift in crop planting and harvesting dates for the last two decades is < 2 weeks^32^ and < 5 days per 1 °C warming^33^.

### Climate data

We obtained daily maximum and minimum air temperatures and daily total precipitation for the period 1958–2013 from a 0.5° global retrospective meteorological forcing dataset called S14FD^34^. Daily mean temperature was derived by averaging the daily maximum and minimum temperatures. For projecting climate, we used the 0.5° statistically downscaled, bias-corrected, daily outputs of eight GCMs that were used in the Coupled Model Intercomparison Project phase 5 (CMIP5^35^ under the four RCPs of 2.6, 4.5, 6.0, and 8.5 W m^−2^^36^ (Supplementary Table 5). The GCM outputs were spatially interpolated onto the 0.5° regular grid coordinate using the inverse distance weighting method and then bias-corrected using the cumulative distribution function (CDF)-based downscaling method^37,38^. S14FD was used as a reference for bias correction^34^. In short, the error in the GCM data for a climatic variable was defined for each percentile of the empirical CDFs derived from the GCM and S14FD for a training period. The defined GCM error was then removed from the empirical CDF of the GCM data for a bias-correction period with the assumption that the error–percentile relationship does not change over time.

Among 32 climate scenarios consisting of eight GCMs and four RCPs, we selected GFDL-ESM2M and HadGEM2-ES under RCP2.6 and RCP8.5 to project changes in area, yield, and production. This selection was based on the relationship between the annual temperature and precipitation changes over the rice area in Asia (Fig. 1a) for the middle (average of 2041–2060) and end (average of 2081–2100) of the twenty-first century projected by eight GCMs and four RCPs, relative to 1986–2005 (Fig. 1b,c). A positive correlation was observed between the projected precipitation and temperature changes (Fig. 1d). HadGEM2-ES under RCP8.5 (r85gH) was the climate scenario that exhibited a larger change along the relationship described above (temperature, + 4.7 °C; and precipitation, + 11.1%), whereas GFDL-ESM2M under RCP2.6 (r26gG) exhibited a smaller change (+ 1.1 °C and + 0.7%). HadGEM2-ES under RCP2.6 (r26gH; + 1.8 °C and + 6.2%) and GFDL-ESM2M under RCP8.5 (g85gG; 2.9 °C and + 7.5%) were scenarios associated with intermediate temperature and precipitation changes. The rice area map in 2005^39^ used as the weights was kept constant in the calculation of average precipitation and temperature changes over the rice area.

### Statistical area and yield models

We established empirical models that link area anomalies with extreme and average precipitation and temperature indices using the elastic net regression technique^40^. Elastic net regression is a general form of a regularized regression model that includes Lasso and Ridge. Since good performance even in out-of-sample validation has been reported for the elastic net regression models^25,26,41^, these models were used. More importantly, the elastic net regression models can be applied in cases where the number of predictors is larger than the number of samples, as well as cases in which predictors were correlated with each other. Due to these features, the model has been used for climate-yield analysis^25,26,41^ and is well suited for this study as some of the climate indices (or the climatic predictors) are correlated with each other, and the number of predictors of 162–243 (= 27 climate indices $\times$ 3 periods per season $\times$ 2–3 seasons) was larger than the sample size of response crop variable of ~ 52 years.

The model for area anomalies was built for each administrative unit. This way of modeling help consider the major characteristics of administrative-unit-level rice response to growing season climate influenced by local management, such as cultivar choice. A single 0.5° grid cell that contained the largest rice area was identified from the rice area map in 2005^39^ and used as the representative location for each administrative unit. We used an approach known as a rolling forecasting origin^42^ to enable us to systematically conduct model development and out-of-sample validation. We built a model using 25 samples from *t − *25 to *t − *1 to predict year *t* and repeated this procedure 25 times for validation. Since the census area and yield data are available for the 52 years, this setting indicates that half of the data was used as the training subset and the remaining half was used as the validation subset. As a result, 27 models were developed from the initial model predicting 1986 (built based on the period 1961–1985) to the last model predicting 2012 (built based on the period 1987–2011). Then, annual time series of the median of 27 predictions was calculated and used for the analysis. The models for the yield anomalies were developed in a similar manner as those for the area anomalies.

The regression coefficients of the elastic net regression models were used to characterize the area and yield responses to climate. The regression coefficients for each administrative unit were automatically standardized within the model-fitting procedure in R and then averaged over the administrative units of country and models with different training subsets and used to characterize average climate influences at the country scale. The characterization was performed separately for area and yield with a focus on the distinction between wet and dry seasons, precipitation and temperature, sowing and growing periods, and extreme and average climates.

### Reliability of predictions

We displayed an indicator of the reliability of projection for projected area, yield, and production. The indicator is the ratio of climatic predictors that fall within the observed min–max ranges when deriving the projection for a given year from the elastic net regression models. The total number of samples used to calculate the ratio for a year is equal to the number of climatic predictors (162–243). The different models produced using different training subsets were not considered to calculate the ratio, since the observed ranges of climatic predictors input to the models are almost identical between the training subsets. The lower the ratio, the more the climatic predictors fall outside the observed min–max ranges and therefore the projection derived from those elastic net regression models are interpreted to be less reliable. The exclusion of unreliable projections (ratio < 90%) decreases the sample size used to test the significance of the slope of a trend line calculated for each of area, yield, and production. As such, the reliability of the projections is built into the trend test. We considered that this treatment is necessary to avoid unrealistically large changes in area, yield, and production, which could occur when inputting unprecedent values of climatic predictors to empirical models, including the elastic net regression models. In addition, such artifacts are more likely to arise in empirical models using extreme climate indices as predictors, compared to models using season average climate indices as predictors.

### Yield projections from process-based models

We obtained rice yield projections for the period 2006–2100, derived using the process-based global gridded crop models (GGCMs) forced by CMIP5 GCMs^43,44^, and compared them with yield projections derived from the elastic net regression models. This dataset is known as the product of the Agricultural Model Intercomparison and Improvement Project (AgMIP, which was part of the Global Gridded Crop Model Intercomparison Initiative (GGCMI^45^, and the Inter-Sectoral Impacts Model Intercomparison Project (ISI-MIP^46^. We used the outputs from four GGCMs (EPIC, GEPIC, IMAGE-AEZ, and LPJmL) forced by HadGEM2-ES and GFDL-ESM2M under RCP2.6 and RCP8.5 to ensure consistency among comparisons. Other GGCMs available in the dataset did not simulate rice^44^. The projected yields were available separately for rainfed and fully irrigated conditions as well as for with and without the fertilization effect from elevated atmospheric carbon dioxide concentration [CO_2_]. We calculated the ensemble mean of the four GGCMs for each of four settings consisting of r26gG/r26gH/r85gG/r85gH, rainfed/irrigation, and with/without the [CO_2_] effect and evaluated whether our regression-based yield projections were plausible considering the known uncertainty in GGCM-based yield projections.

## Supplementary Information

Supplementary Information.

## Data Availability

All data supporting the analysis performed in this study are publicly available from open sources. The rice census data are accessible from statistical year books of the individual country. The S14FD meteorological forcing dataset is available at https://doi.org/10.20783/DIAS.523. The bias-corrected CMIP5 GCM outputs can be obtained from https://doi.org/10.20783/DIAS.524.
