# Supplementary material for: Contrasting area and yield responses to extreme climate contributes to climate-resilient rice production in Asia
Source: Sci Rep. 2023 Apr 17;13:6219. doi: 10.1038/s41598-023-33413-7 (PMC10110525; doi:10.1038/s41598-023-33413-7)
Supplement: Supplementary file 1 — Supplementary Information. [file 41598_2023_33413_MOESM1_ESM.docx]

Supplementary Information

**Comparisons between regression- and GGCM-based yield projections**

We found that the spread of GGCM-based projections is considerably large, and the regression-based yield projections favorably well fall within the spread of multi-GGCM mean projections with different settings on irrigation and [CO_2_] effect. The regression-based projections did not consider the [CO_2_] effect. However, there was no such tendency that the regression-based projections appeared to be close to the GGCM-based projections without the [CO_2_] effect (Supplementary Fig. 9).

Supplementary Table 1. Summary of rice seasons conducted in the studied countries.

| Country | Number of subnational units studied | Rice season and cropping system^1^ | Rice calendar^2^ |
| --- | --- | --- | --- |
| Bangladesh | 21 | 3: Aman (r), Aus (r), Boro (i) | GIEWS |
| Indonesia | 34 | 2: Main (r), Second (i) | AMIS |
| Malaysia | 13 | 2: Main (r), Second (i) | GIEWS |
| Myanmar | 14 | 2: Main (r), Second (i) | GIEWS |
| Philippines | 71 | 2: Wet (r), Dry (i) | AMIS |
| Thailand | 1 | 2: Main (r), Second (i) | AMIS |
| ^1^ r, rainfed; i, irrigated.  ^2^ GIEWS, http://www.fao.org/giews/countrybrief/index.jsp; AMIS, http://www.amis-outlook.org/amis-about/calendars/maizecal/en/ | | | |

Supplementary Table 2. Inter-quantile ranges (IQRs) of area, yield, and production anomalies and the correlation between area and yield anomalies. The data are based on the census reports for the period 1987–2012 (n = 26).

| Country | IQR (%) | | | Correlation between area and yield | |
| --- | --- | --- | --- | --- | --- |
|  | Area | Yield | Production | R | p-value |
| Bangladesh | 4.8 | 5.2 | 7.5 | 0.530 | 0.005 |
| Indonesia | 4.9 | 12.5 | 15.1 | 0.095 | 0.643 |
| Malaysia | 3.3 | 7.4 | 6.0 | -0.066 | 0.749 |
| Myanmar | 5.8 | 5.2 | 9.3 | 0.480 | 0.013 |
| Philippines | 5.0 | 4.7 | 6.0 | 0.161 | 0.433 |
| Thailand | 5.9 | 11.6 | 12.3 | -0.124 | 0.545 |

Supplementary Table 3. Top contributing climatic predictors identified based on the regression coefficients of the elastic net regression models. +, positive effect; –, negative effect. Suffix: .s, sowing period; .g, growing period (the average of early and late growing period groups). See also Supplementary Table 4.

| Bangladesh  Area–Temperature   - Wet/rainfed: +) TNn.s, SU.s, TXn.s; –) TN10p.s, TX10p.s, CSDI.g - Dry/irrigated: +) CSDI.s, TXn.s, TXn.g; –) CSDI.g, TXx.g, TNx.g   Area–Precipitation   - Wet/rainfed: +) CDD.s, Rx1day.s, R99pTOT.s; –) R99pTOT.g, R95pTOT.s, R95pTOT.g - Dry/irrigated: +) R20mm.g, R10mm.g, CWD.g; –) R99pTOT.g, CDD.s, CDD.g   Yield–Temperature   - Wet/rainfed: +) TXn.s, TN10p.s, SU.s; –) TNx.s, TNx.g, DTR.s - Dry/irrigated: +) TNx.s, TNn.s, TXx.s; –) TNx.g, DTR.g, TXx.g   Yield–Precipitation   - Wet/rainfed: +) PRCPTOT.s, RM.s, Rx1day.s; –) SDII.g, CWD.s, R95pTOT.g - Dry/irrigated: +) Rx1day.s, R10mm.s, Rx5day.s; –) Rx1day.g, SDII.g, CWD.s |
| --- |
| Indonesia  Area–Temperature   - Wet/rainfed: +) TNn.g, TR.s, TX10p.s; –) SU.g, TX90p.g, WSDI.s - Dry/irrigated: +) TNn.g, TR.g, TNx.g; –) DTR.s, WSDI.s, DTR.g   Area–Precipitation   - Wet/rainfed: +) CWD.s, R10mm.s, PRCPTOT.s; –) CDD.s, Rx1day.s, R99pTOT.s - Dry/irrigated: +) R10mm.g, PRCPTOT.g, RM.g; –) CDD.g, CDD.s, R95pTOT.s   Yield–Temperature   - Wet/rainfed: +) TXn.g, TN90p.g, TNn.s; –) TX10p.g, TX90p.s, TX90p.g - Dry/irrigated: +) SU.s, TXn.s, TXn.g; –) TX10p.s, TN10p.s, TX10p.g   Yield–Precipitation   - Wet/rainfed: +) RM.s, PRCPTOT.s, SDII.s; –) CWD.s, CDD.s, R95pTOT.s - Dry/irrigated: +) CDD.g, Rx5day.g, CWD.s; –) R10mm.g, R99pTOT.s, R95pTOT.g |
| Malaysia  Area–Temperature   - Wet/rainfed: +) SU.s, TXn.g, TX10p.s, -) TXx.g, TR.s, TNx.s - Dry/irrigated: +) TXn.g, TN10p.g, TXn.s, –) TX90p.g, TXx.s, TXx.g   Area–Precipitation   - Wet/rainfed: +) CDD.s, R95pTOT.g, R99pTOT.g, –) R20mm.g, CWD.g, CDD.g - Dry/irrigated: +) R99pTOT.g, R99pTOT.s, R95pTOT.g, –) CWD.g, Rx5day.s, R10mm.g   Yield–Temperature   - Wet/rainfed: +) TX90p.s, WSDI.s, TX10p.g, –) TNn.g, TX90p.g, TXx.g - Dry/irrigated: +) TXn.g, WSDI.g, WSDI.s, –) TXn.s, TXx.s, TX10p.g   Yield–Precipitation   - Wet/rainfed: +) CDD.g, R99pTOT.s, Rx1day.s,–) R10mm.g, R10mm.s, R20mm.g - Dry/irrigated: +) CWD.s, CDD.s, Rx5day.g, –) R10mm.s, R20mm.g, R95pTOT.g |
| Supplementary Table 3. (continued) |
| Myanmar  Area–Temperature   - Wet/rainfed: +) TNn.s, CSDI.s, TN10p.s, –) DTR.g, WSDI.g, TNx.g - Dry/irrigated: +) TXx.s, TX10p.s, TXn.g, –) SU.g, TN90p.s, TNx.g   Area–Precipitation   - Wet/rainfed: +) CDD.s, R10mm.g, R99pTOT.g, –) CDD.g, R99pTOT.s, R95pTOT.s, - Dry/irrigated: +) CWD.s, R99pTOT.s, Rx1day.g, –) CDD.s, R10mm.s, R95pTOT.s   Yield–Temperature   - Wet/rainfed: +) TNn.s, TNx.s, TNn.g, –) TX10p.s, TN10p.s, TX90p.g - Dry/irrigated: +) TX10p.s, TXx.s, TNx.g, –) WSDI.g, TX10p.g, TX90p.g   Yield–Precipitation   - Wet/rainfed: +) R10mm.s, CWD.g, CDD.s, –) R99pTOT.g, Rx1day.s, R99pTOT.s, - Dry/irrigated: +) SDII.s, R95pTOT.s, R10mm.s, –) CWD.g, CWD.s, R20mm.g |
| Philippines  Area–Temperature   - Wet/rainfed: +) TX10p.s, TX10p.g, TNn.s, –) DTR.s, TXn.s, TX90p.s - Dry/irrigated: +) TX10p.g, TX10p.s, TNx.g, –) TNn.s, TXx.g, SU.s,   Area–Precipitation   - Wet/rainfed: R10mm.s, CWD.s, PRCPTOT.s, –) CDD.s, CDD.g, SDII.s - Dry/irrigated: +) CDD.g, R95pTOT.s, CDD.s, –) R10mm.g, R20mm.g, R20mm.s   Yield–Temperature   - Wet/rainfed: +) TR.s, DTR.s, TNx.s, –) CSDI.s, TX10p.s, SU.s - Dry/irrigated: +) TXx.s, TXx.g, TX90p.g, –) TNn.g, TX10p.g, TNx.g   Yield–Precipitation   - Wet/rainfed: +) SDII.s, CDD.s, R95pTOT.s, –) R99pTOT.s, Rx1day.s, Rx5day.s - Dry/irrigated: +) R10mm.g, R95pTOT.g, CDD.s, –) CWD.g, CDD.g, CWD.s |
| Thailand  Area–Temperature   - Wet/rainfed: +) TXn.g, DTR.s, TXx.s, –) WSDI.s, TNx.s, TR.g - Dry/irrigated: +) TXx.s, TNx.s, TX10p.g, –) WSDI.s, TN90p.g, SU.g   Area–Precipitation   - Wet/rainfed: +) R20mm.s, SDII.s, RM.s, –) R95pTOT.s, CDD.s, CWD.g - Dry/irrigated: +) R99pTOT.g, CWD.s, Rx5day.s, –) R99pTOT.s, CWD.g, R10mm.s   Yield–Temperature   - Wet/rainfed: +) TXx.g, TX10p.g, WSDI.g, –) TX10p.s, TXx.s, SU.g - Dry/irrigated: +) WSDI.s, TXx.s, TX10p.s, –) CSDI.g, TXn.g, TXn.s   Yield–Precipitation   - Wet/rainfed: +) CDD.g, SDII.s, R95pTOT.s, –) CWD.s, CWD.g, R20mm.g - Dry/irrigated: +) Rx5day.s, CDD.g, R95pTOT.g, –) Rx1day.s, SDII.s, R99pTOT.g |

Supplementary Table 4. List of the extreme and average climate indices. All indices listed here are calculated for three periods (the sowing, early growing group, and late growing group) of each season (wet and dry) described in Supplementary Fig. 1.

| Category^1^ | | Label | Name | Definition | Units | |
| --- | --- | --- | --- | --- | --- | --- |
| T | E | TXx | Hottest day | Maximum daily maximum temperature in the period (T_max_) | °C | |
|  |  | TNx | Warmest night | Maximum daily minimum temperature in the period (T_min_) | °C | |
|  |  | TXn | Coldest day | Minimum T_max_ in the period | °C | |
|  |  | TNn | Coldest night | Minimum T_min_ in the period | °C | |
|  |  | TN10p | Cool nights | Percentage of time when T_min_ < 10th percentile | % | |
|  |  | TX10p | Cool days | Percentage of time when T_max_ < 10th percentile | % | |
|  |  | TN90p | Warm nights | Percentage of time when T_min_ > 90th percentile | % | |
|  |  | TX90p | Warm days | Percentage of time when T_max_ > 90th percentile | % | |
|  |  | DTR | Diurnal temperature range | Mean difference between T_max_ and T_min_ in the period | °C | |
|  |  | ID | Icing days | Count when T_max_ < 0 °C in the period | days | |
|  |  | FD | Frost days | Count when T_min_ < 0 °C in the period | days | |
|  |  | SU | Summer days | Count when T_max_ > 25 °C in the period | days | |
|  |  | TR | Tropical nights | Count when T_min_ > 20 °C in the period | days | |
|  |  | WSDI | Warm spell duration index | Count of at least six consecutive days of T_max_ > 90th percentile in the period | days | |
|  |  | CSDI | Cold spell duration index | Count of at least six consecutive days of T_min_ < 10th percentile in the period | days | |
|  | A | TM | Mean temperature | Mean temperature for the period | °C | |
| Supplementary Table 4. (continued) | | | | | |  |
| P | E | Rx1day | Maximum 1-day precipitation amount | Maximum 1-day precipitation during the period | mm | |
|  |  | Rx5day | Maximum 5-day precipitation amount | Maximum consecutive 5-day precipitation during the period | mm | |
|  |  | SDII | Simple daily intensity index | The ratio of total precipitation to the number of wet days (≥1 mm) during the period | mm | |
|  |  | R10mm | Number of heavy precipitation days | Count of daily precipitation (Pr) ≥10 mm during the period | days | |
|  |  | R20mm | Number of very heavy precipitation days | Count of Pr >=20 mm during the period | days | |
|  |  | CDD | Consecutive dry days | Maximum number of consecutive days of Pr < 1 mm | days | |
|  |  | CWD | Consecutive wet days | Maximum number of consecutive days of Pr ≥ 1mm | days | |
|  |  | PRCPTOT | Total wet day precipitation | Total precipitation in wet days (≥ 1 mm) during the period | mm | |
|  |  | R95pTOT | Contribution from very wet days | The ratio of total precipitation from days with Pr > 95th percentile during the period to PRCPTOT | % | |
|  |  | R99pTOT | Contribution from extremely wet days | The ratio of total precipitation from days with Pr > 99^th^ percentile during the period to PRCPTOT | % | |
|  | A | RM | Mean precipitation | Mean total precipitation for the period | mm | |
| ^1^ T: temperature, P: precipitation, E: extreme index, and A: average index | | | | | |  |

Supplementary Table 5. List of the GCMs and modeling groups obtained from the CMIP5 multi-model ensemble dataset for this study.

| GCM name | Modeling group |
| --- | --- |
| GFDL-ESM2M | NOAA Geophysical Fluid Dynamics Laboratory |
| IPSL-CM5A-LR | Institut Pierre-Simon Laplace |
| HadGEM2-ES | Met Office Hadley Centre |
| MIROC-ESM | Japan Agency for Marine-Earth Science and Technology (JAMSTEC), Atmosphere and Ocean Research Institute/The University of Tokyo (AORI), and National Institute for Environmental Studies (NIES) |
| MIROC-ESMCHEM |  |
| MIROC5 | AORI, NIES and JAMSTEC |
| NorESM1-M | Norwegian Climate Centre |
| MRI-CGCM3 | Meteorological Research Institute |


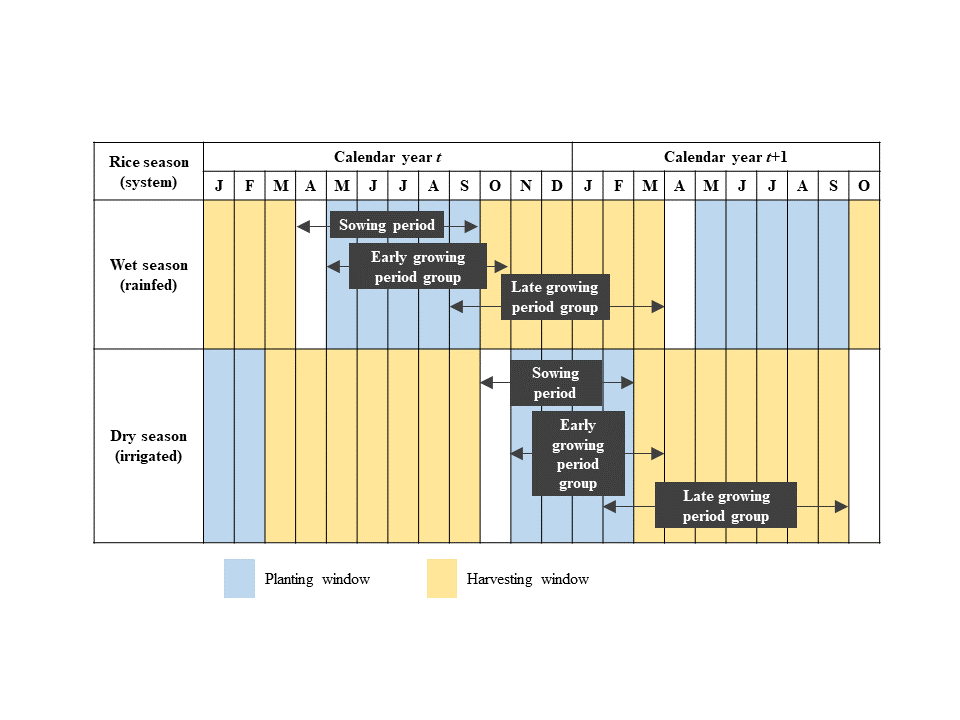


Supplementary Figure 1. Sowing period and two growing periods in the wet and dry seasons. Rice calendars for Thailand are provided for reference.


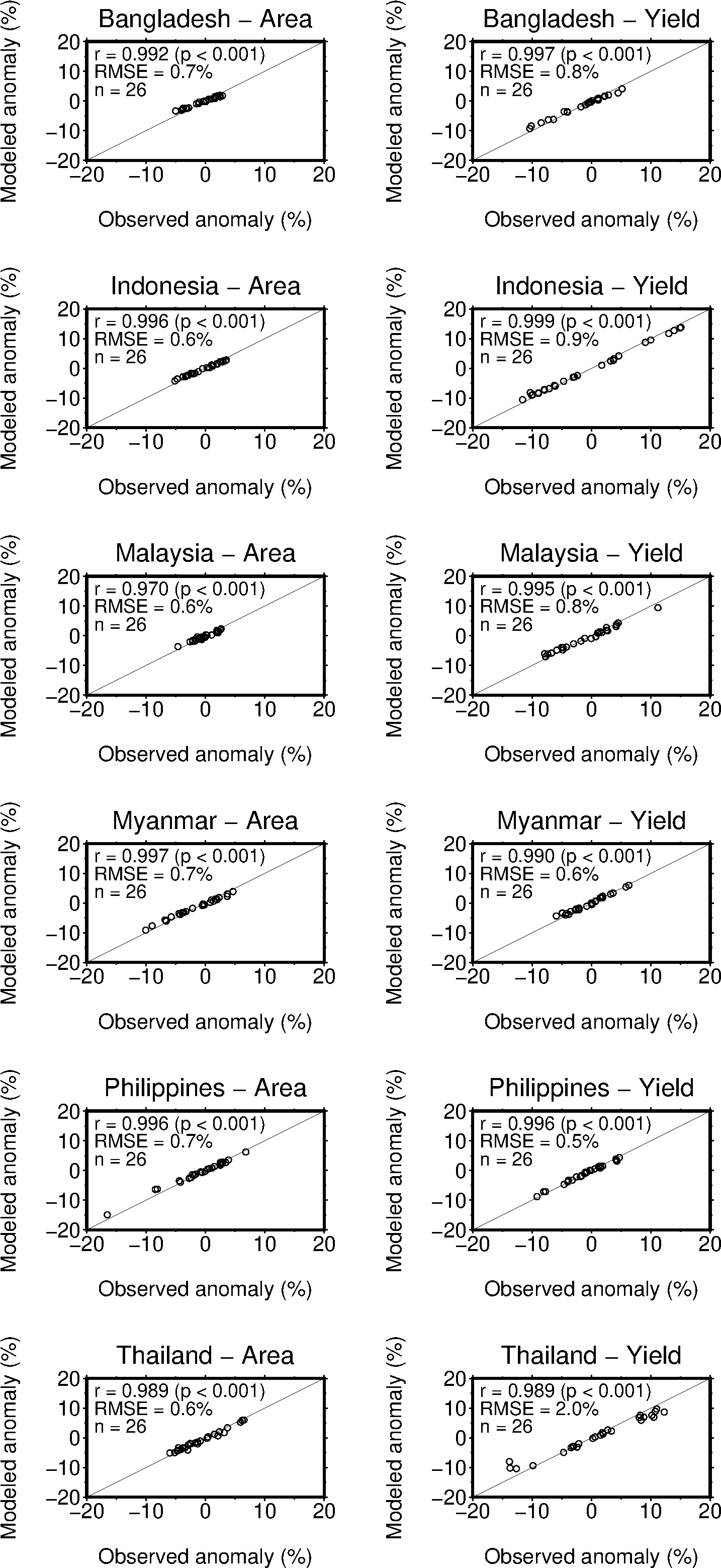


Supplementary Figure 2. Comparisons between the out-of-sample reproduction using the elastic net regression models and reported data for area and yield.


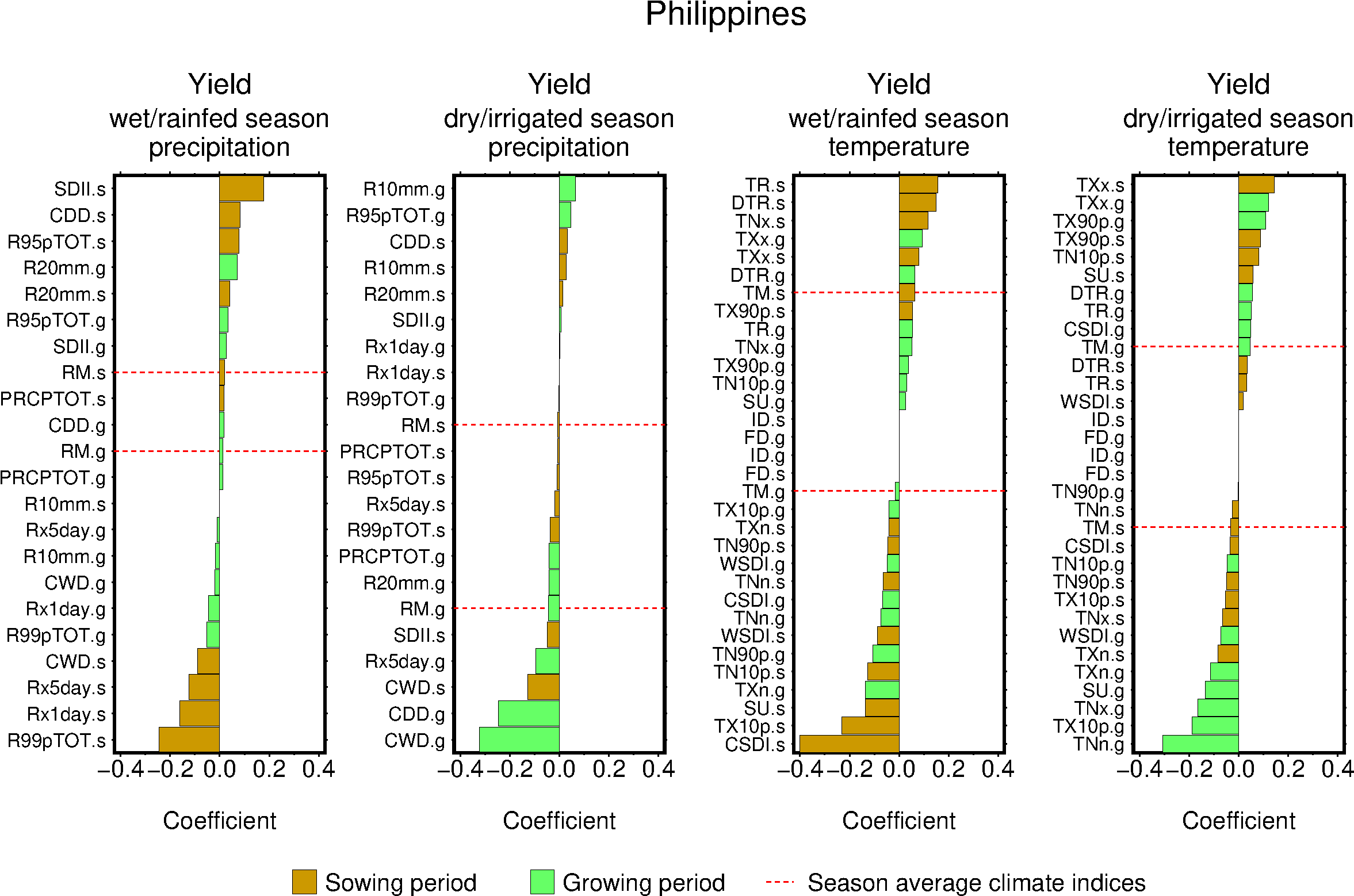


Supplementary Figure 3. Yield response to climate in the Philippines. The values indicate the standardized average regression coefficients of the elastic net regression models. The predictors with and without red dotted lines denote average and extreme climate indices, respectively. Brown bars show the sowing period; Green bars show the growing period of the average early and late groups. The four panels show the seasons (wet/rainfed and dry/irrigated) and climate variables (precipitation and temperature).


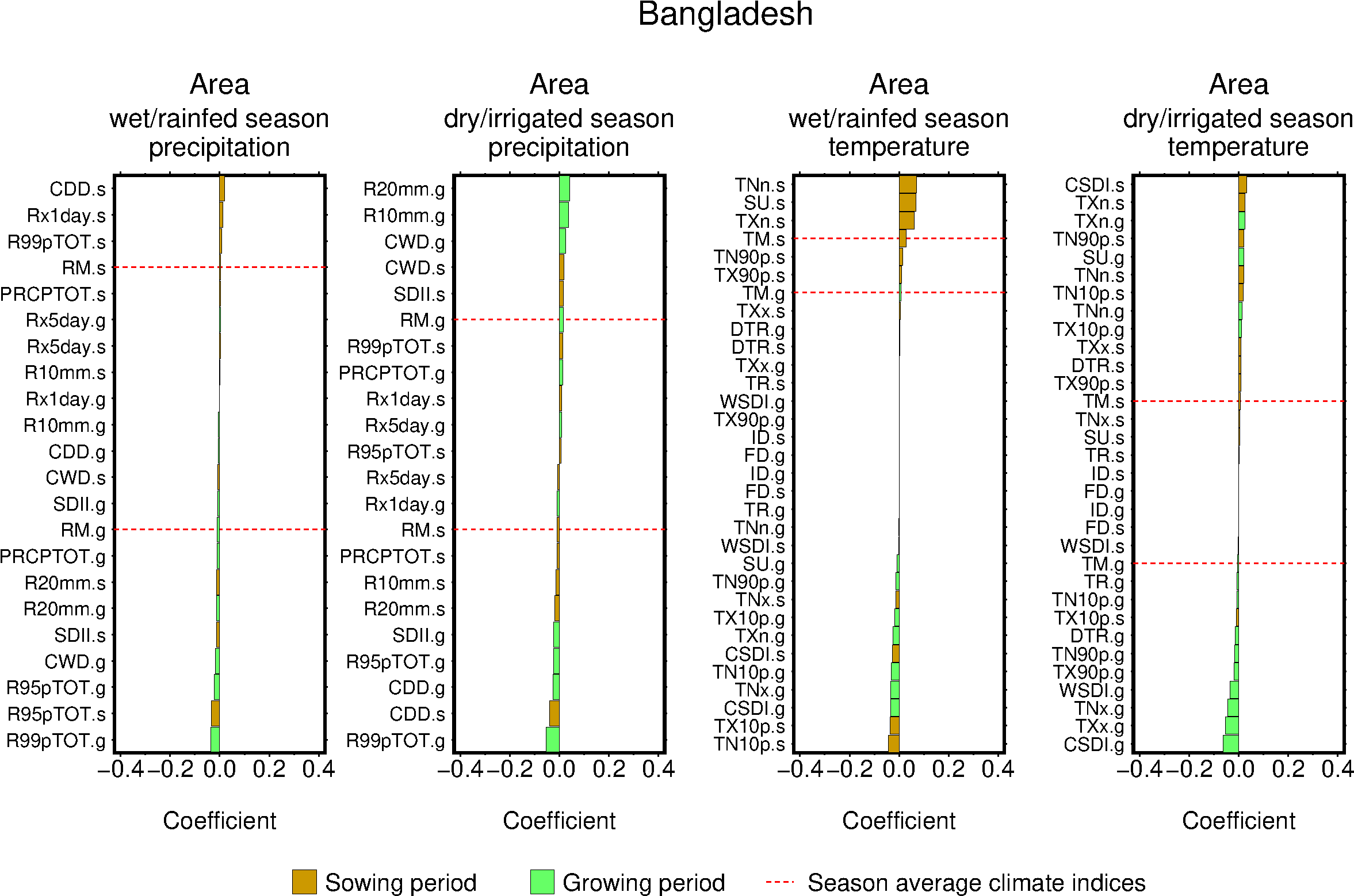


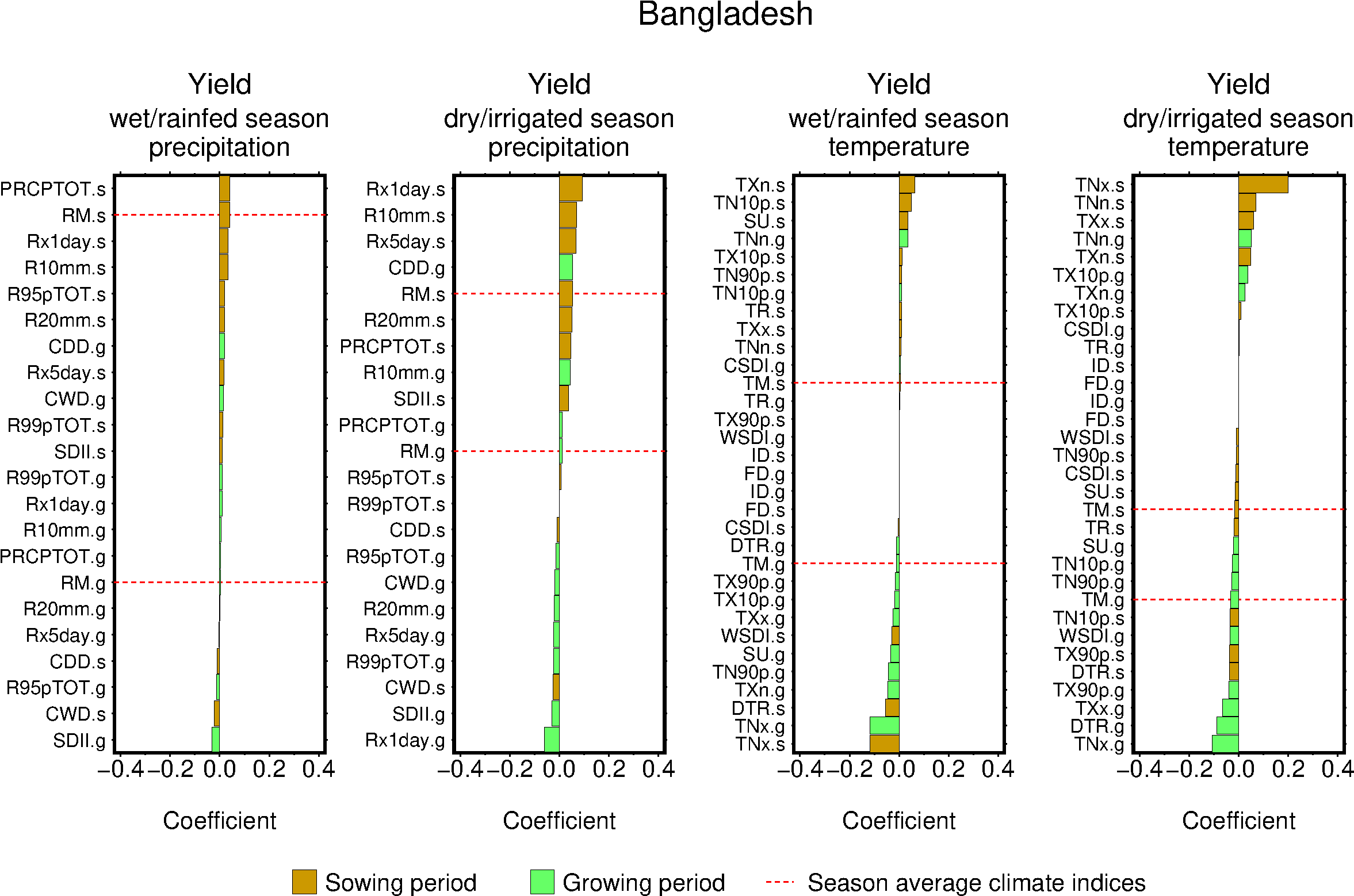


Supplementary Figure 4. Area and yield response to climate in the studied countries other than the Philippines.


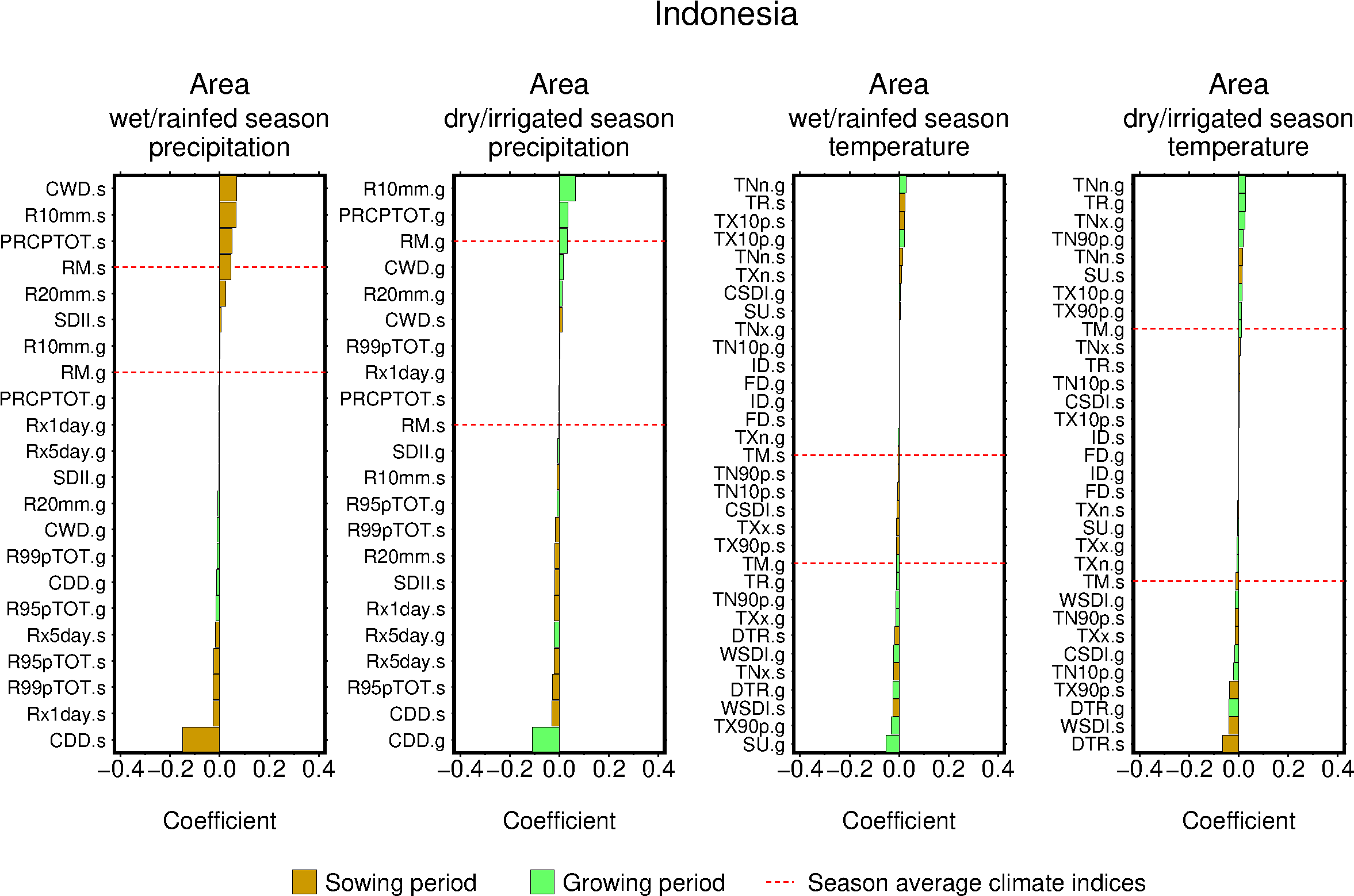


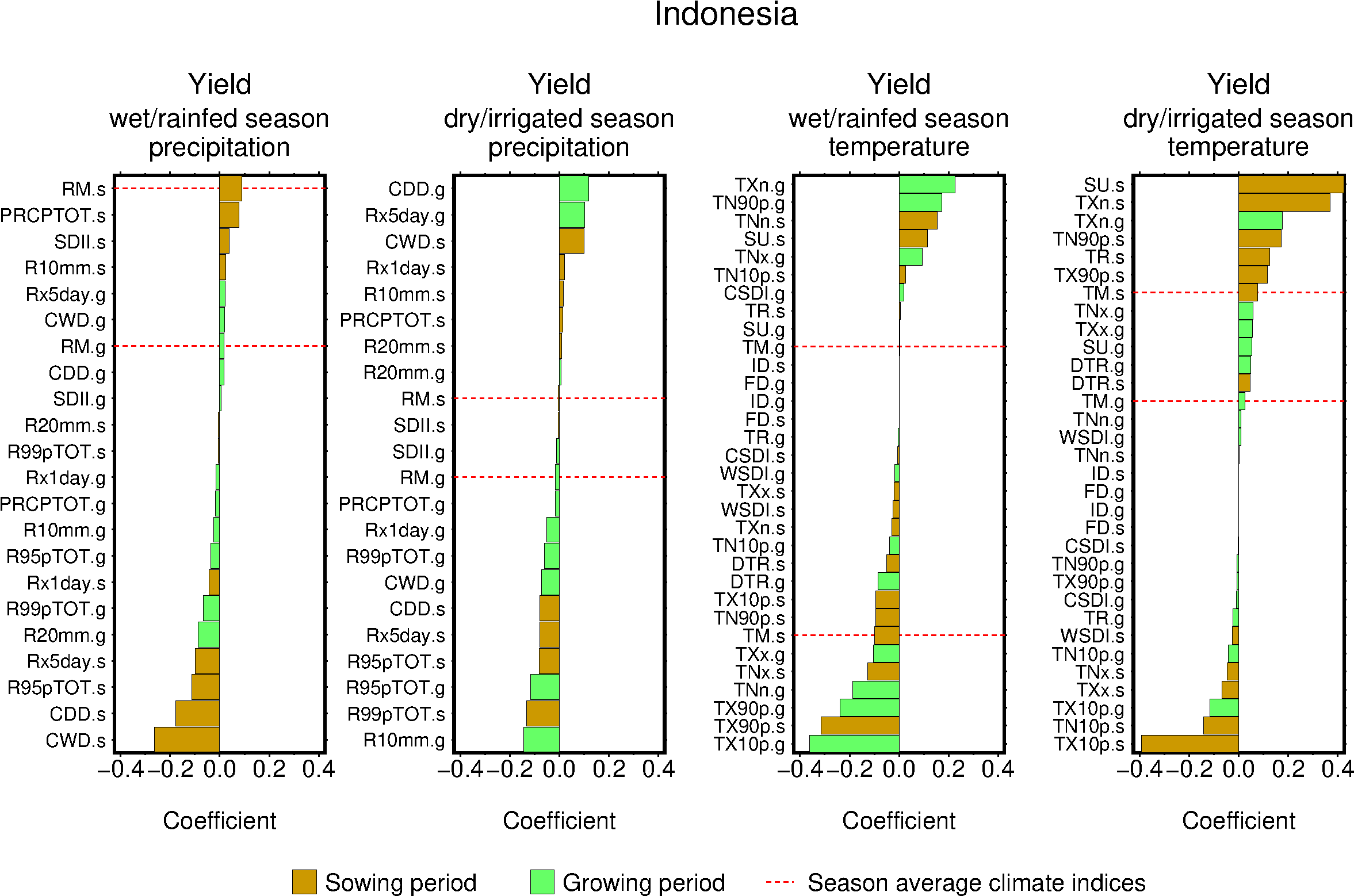


Supplementary Figure 4. (continued)


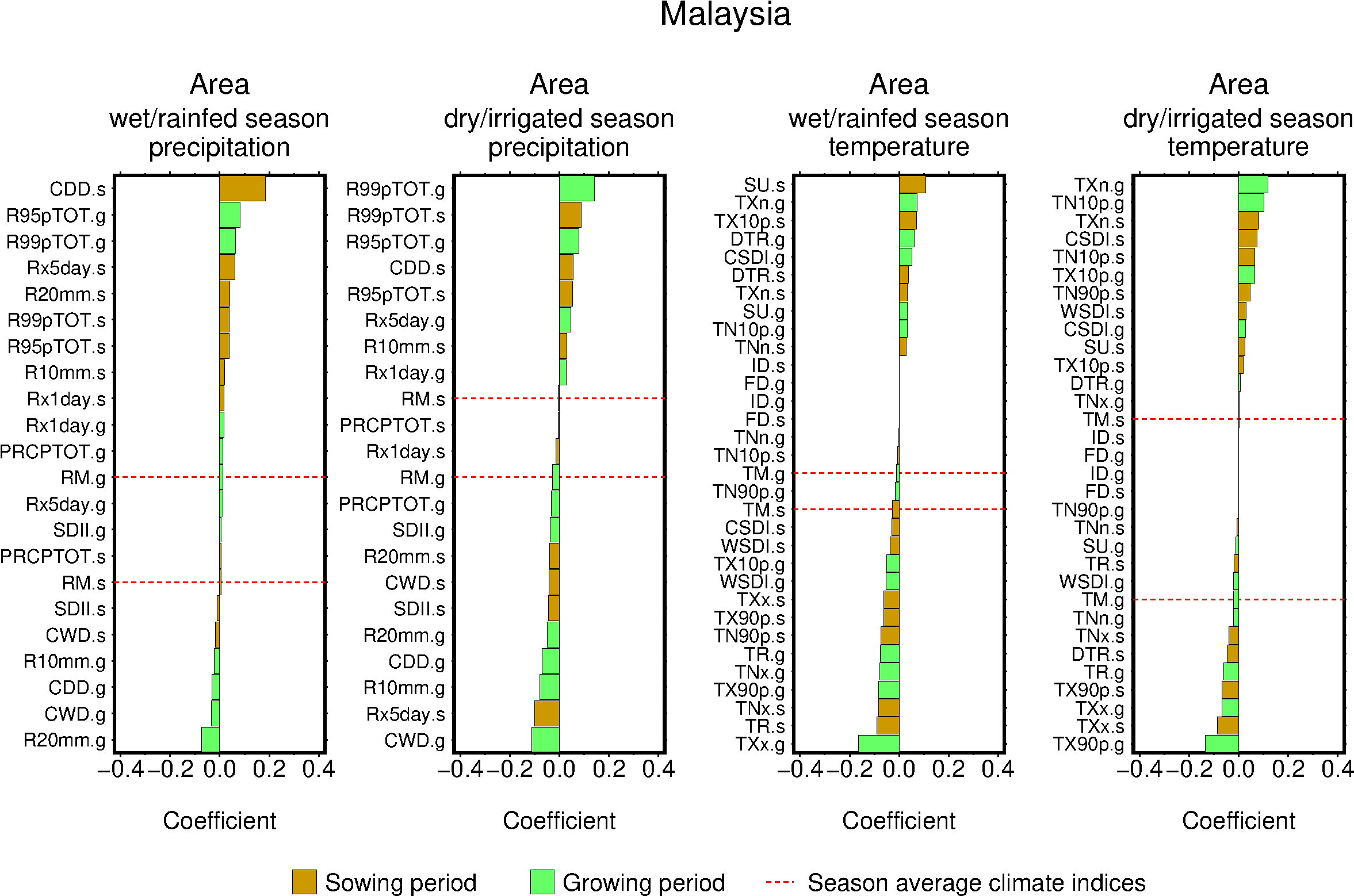


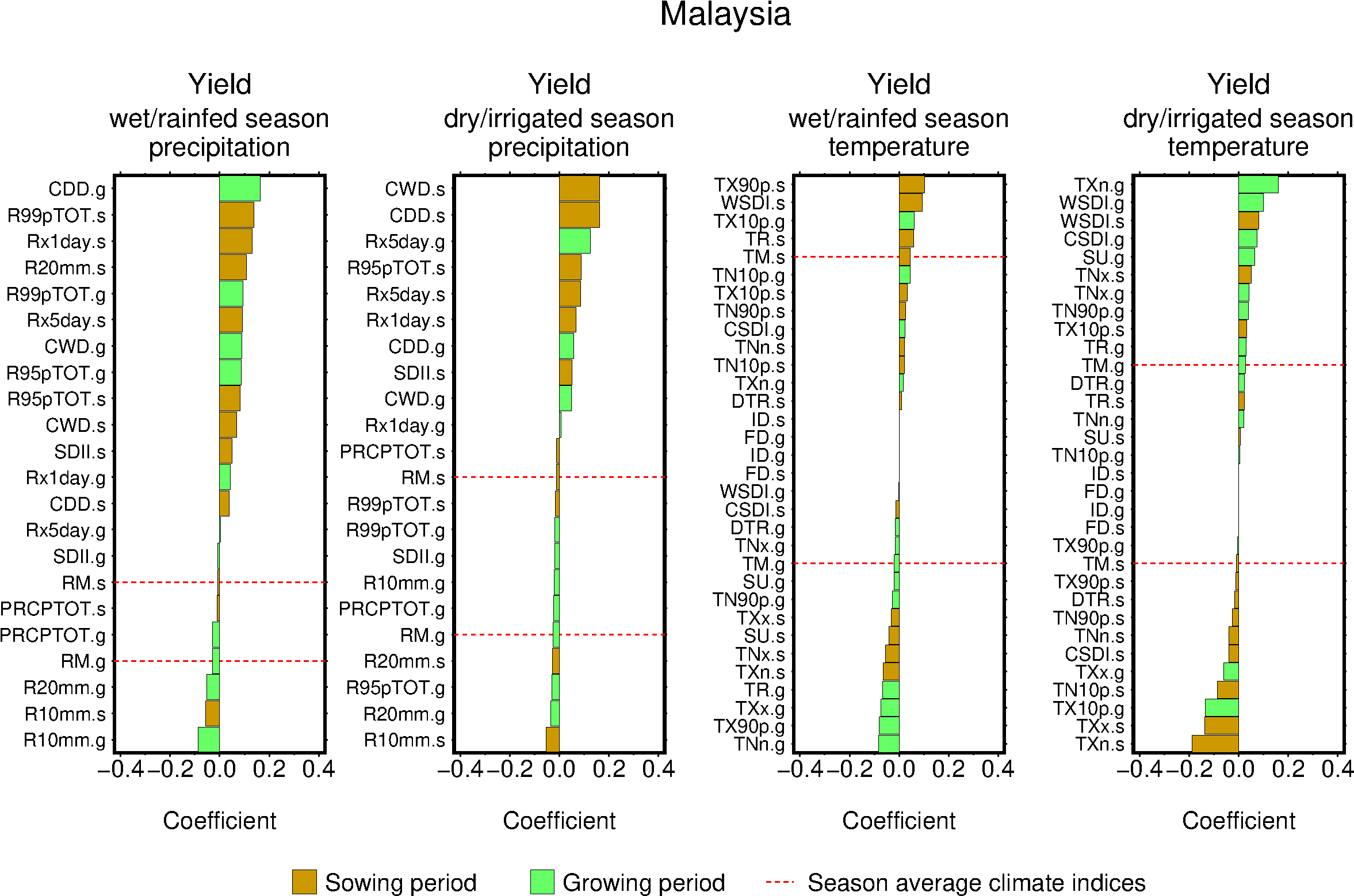


Supplementary Figure 4. (continued)


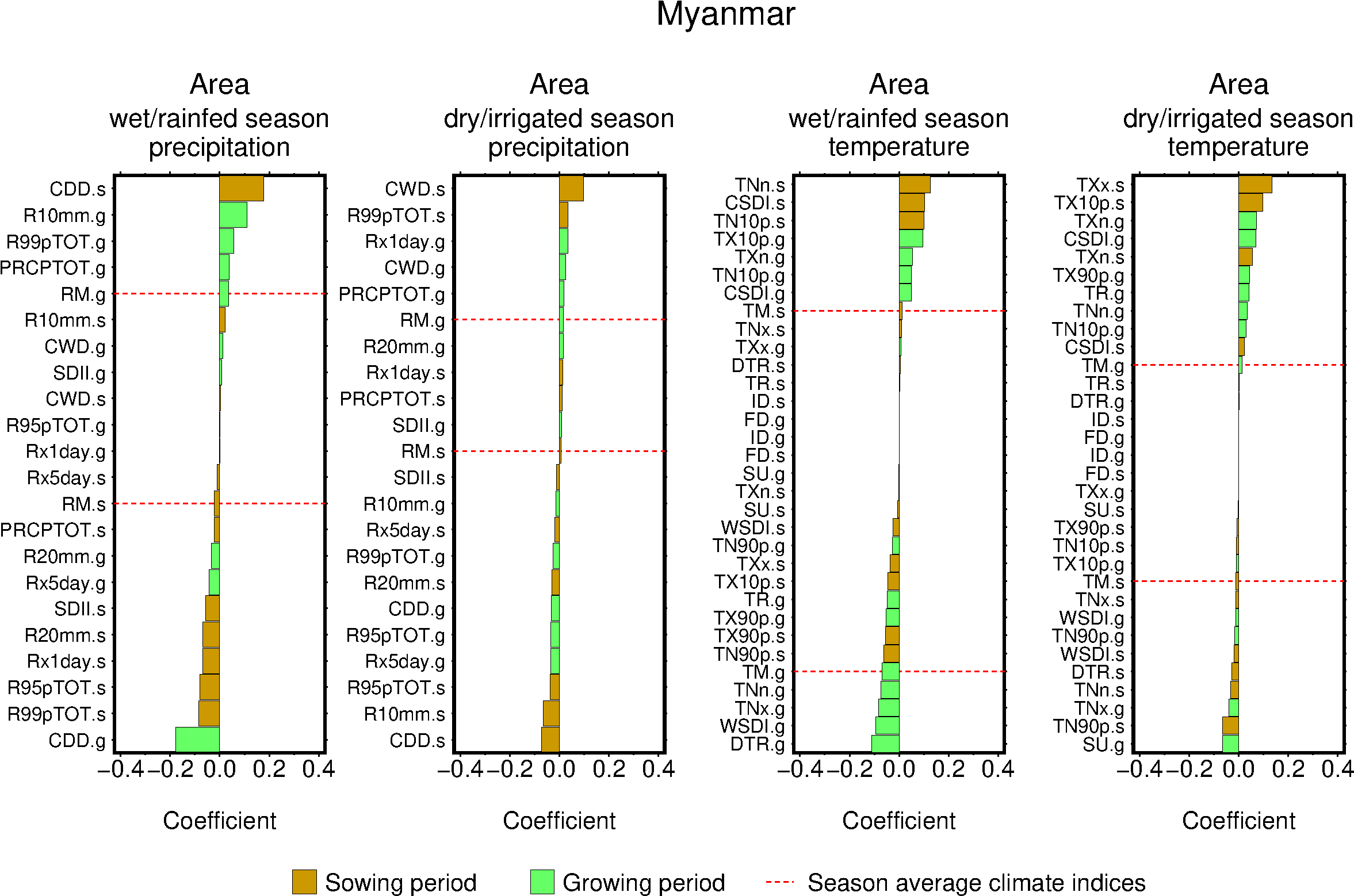


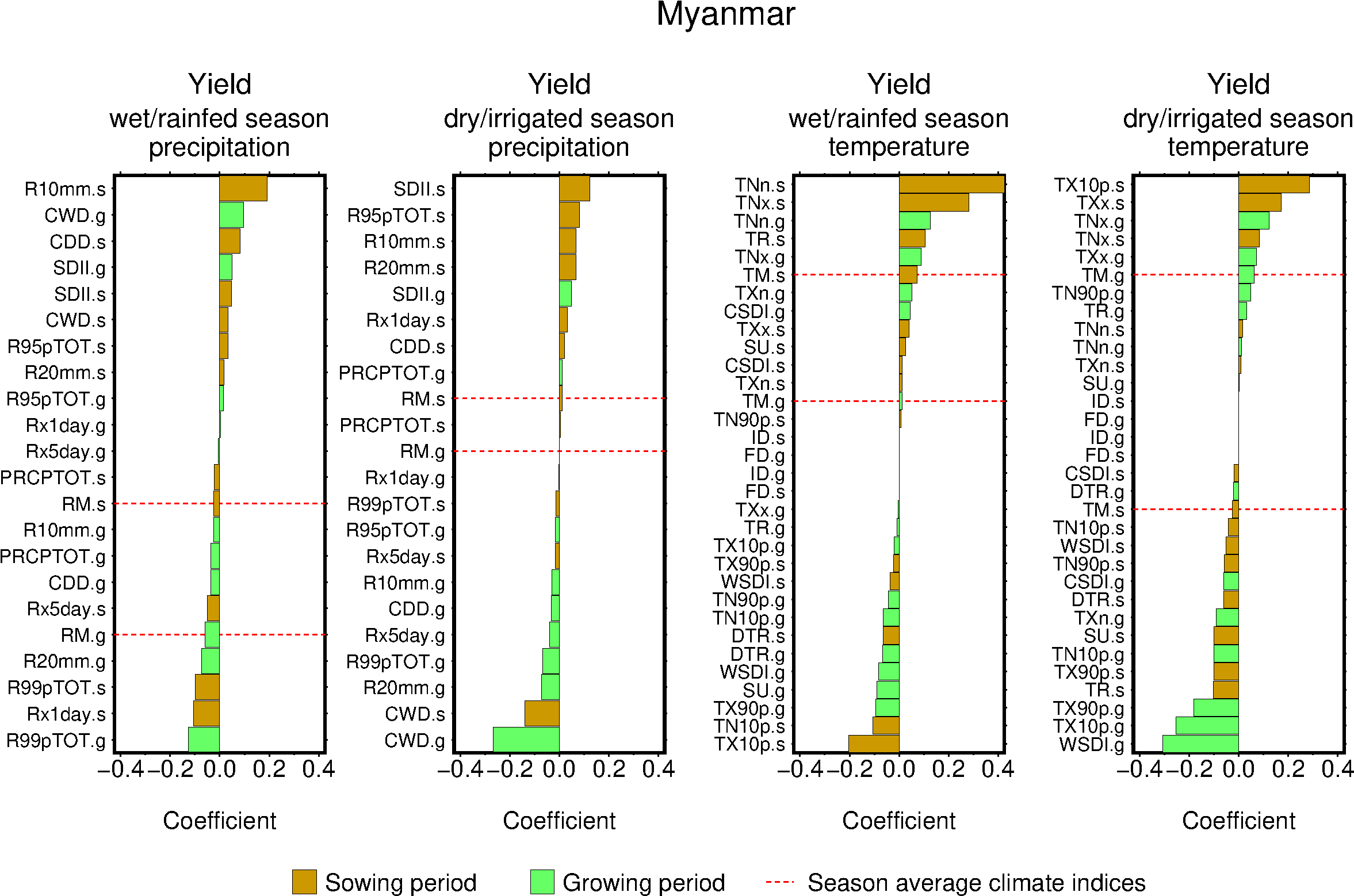


Supplementary Figure 4. (continued)


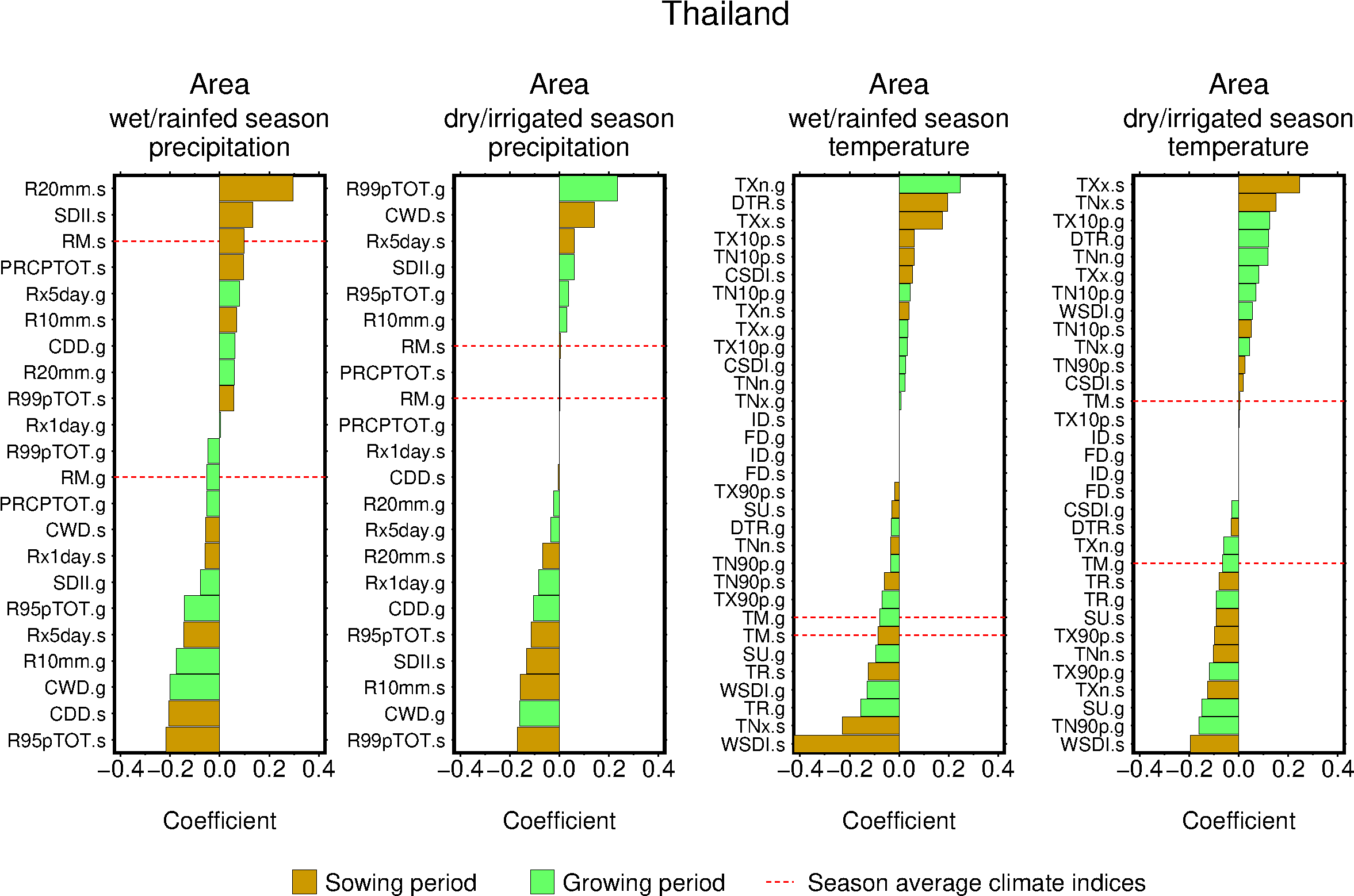


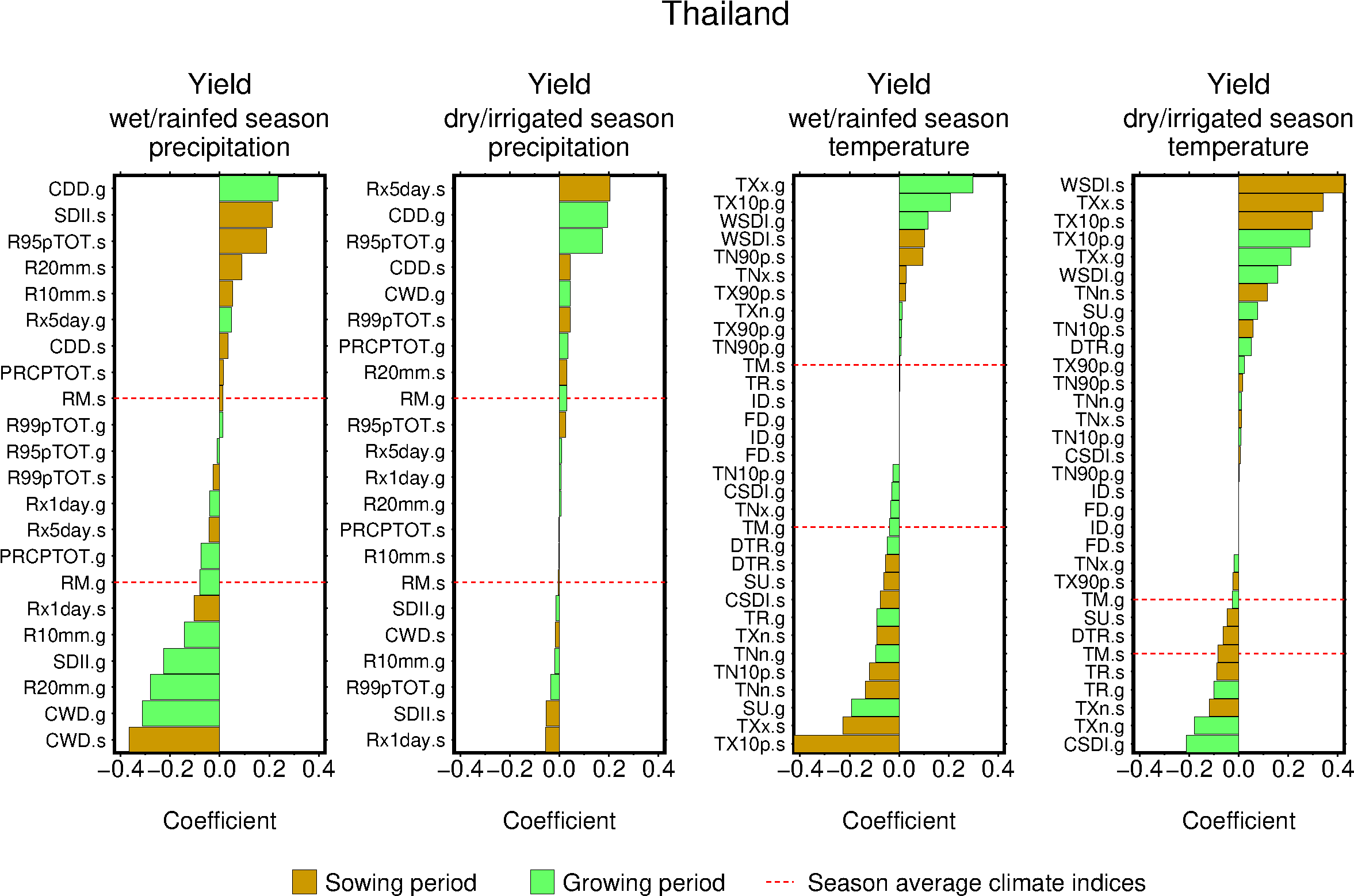


Supplementary Figure 4. (continued)

Supplementary Figure 5. Heat maps showing the counts of top three climatic contributors (for each sign) of area and yield for wet and dry seasons.


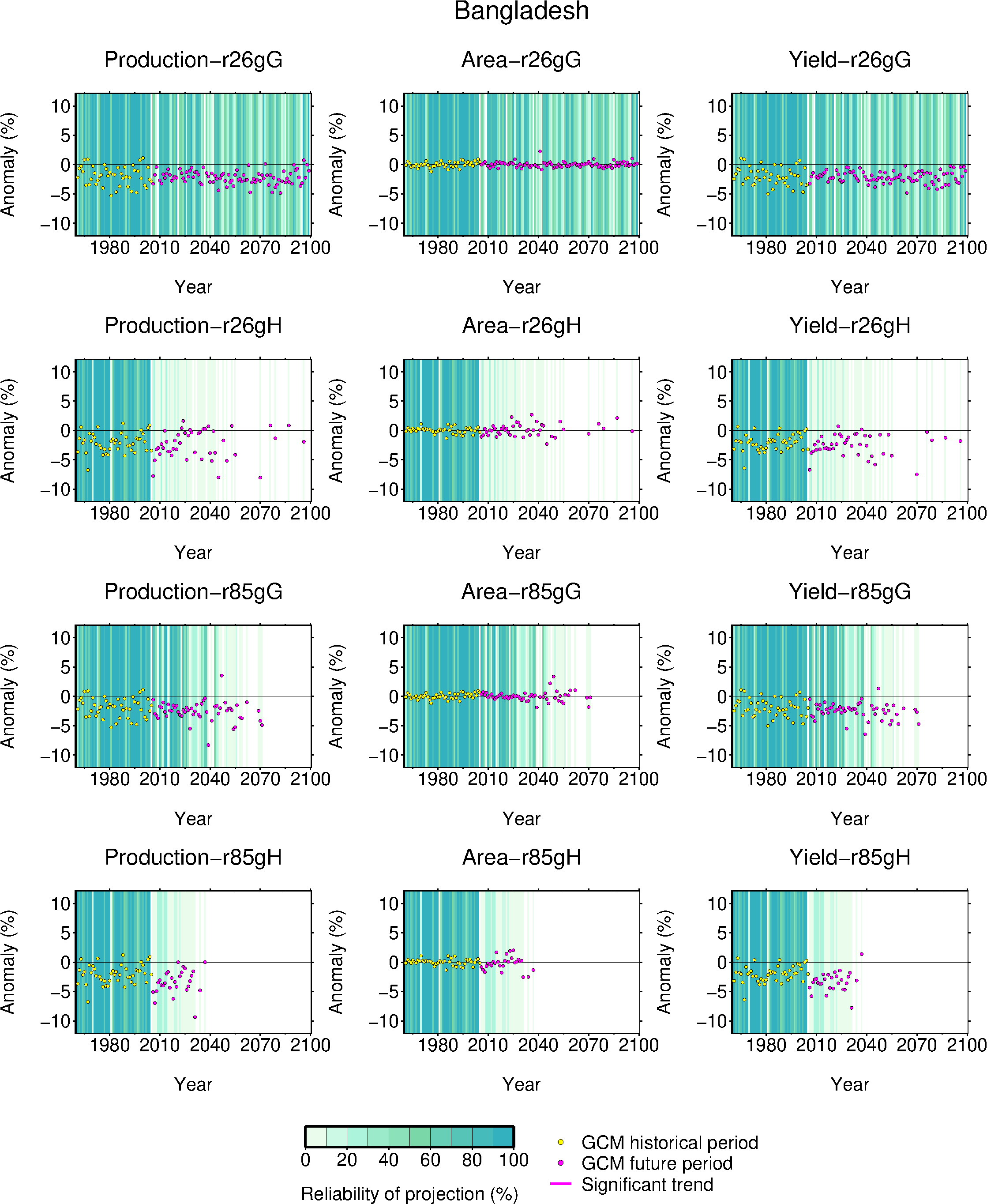


Supplementary Figure 6. Projected changes in production, area, and yield for the studied countries. The heat map overlayed on each panel indicates the reliability of the projection. Lighter green and white columns indicate that many more climatic predictors used to derive that projection fell outside the observed minimum-to-maximum ranges and therefore is less reliable than projections in the darker green column. Less reliable projections are eliminated when fitting a trend line and testing the significance of the slope. Trend lines are shown only when a significant slope is obtained.


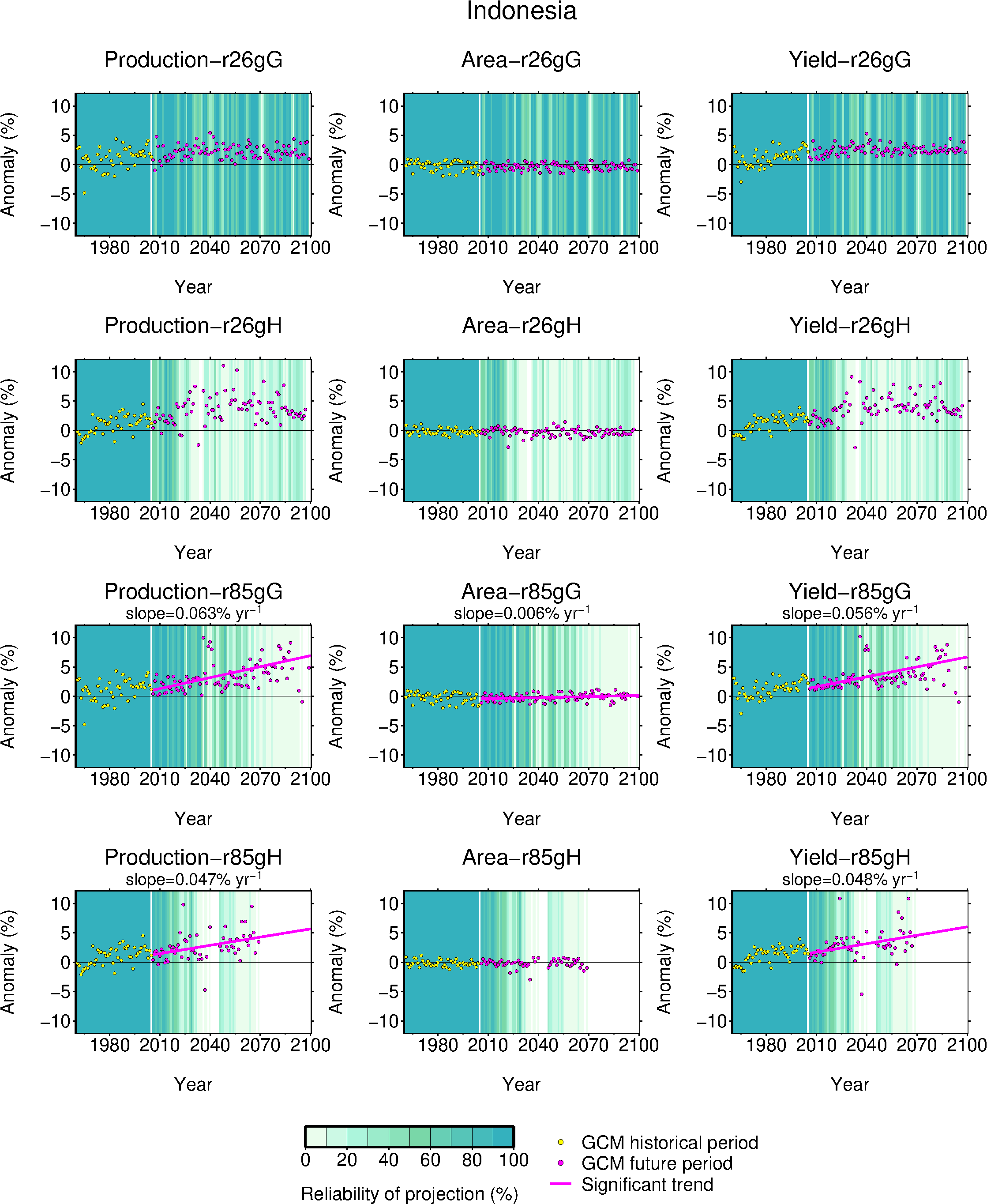


Supplementary Figure 6. (continued)


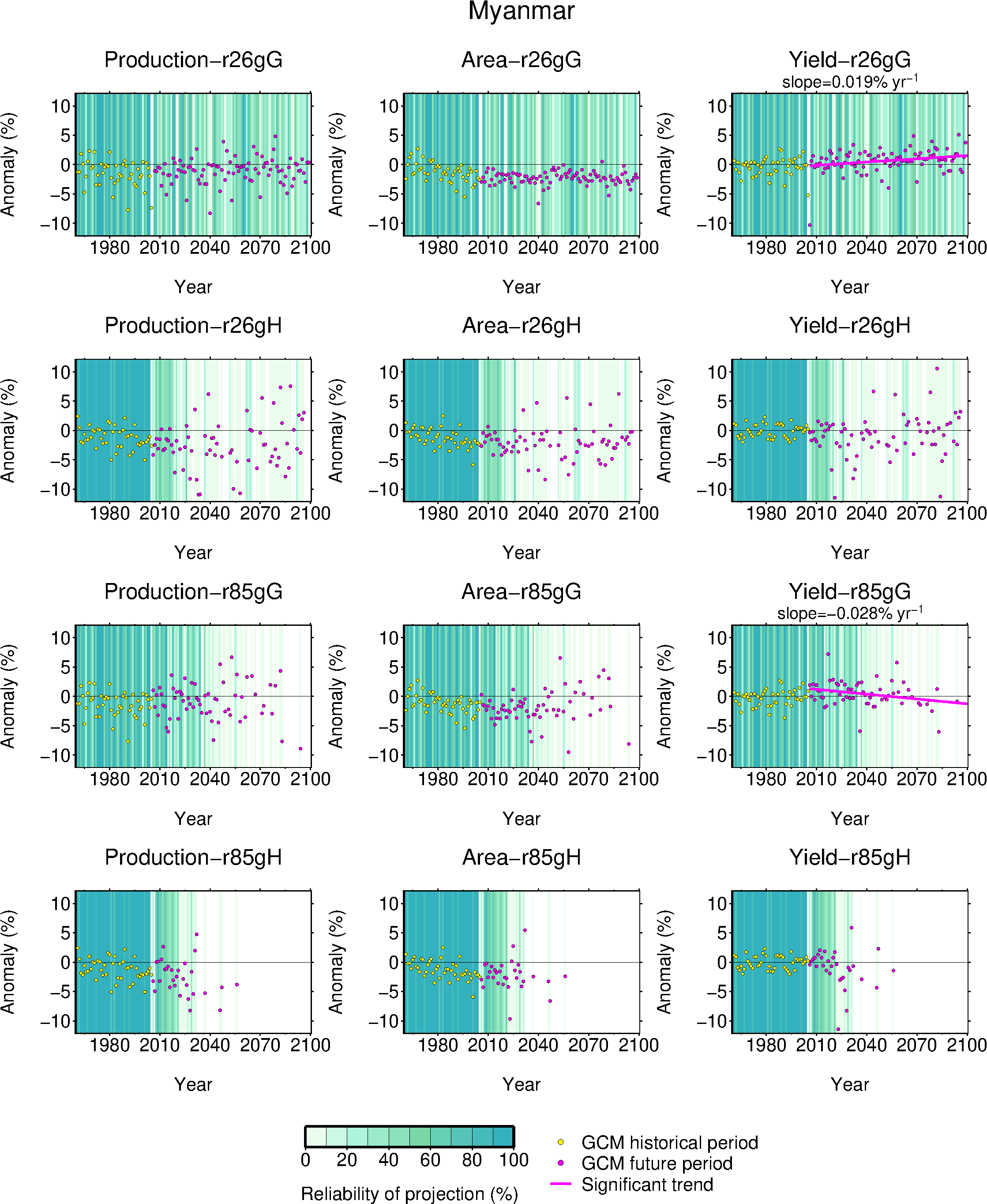


Supplementary Figure 6. (continued)


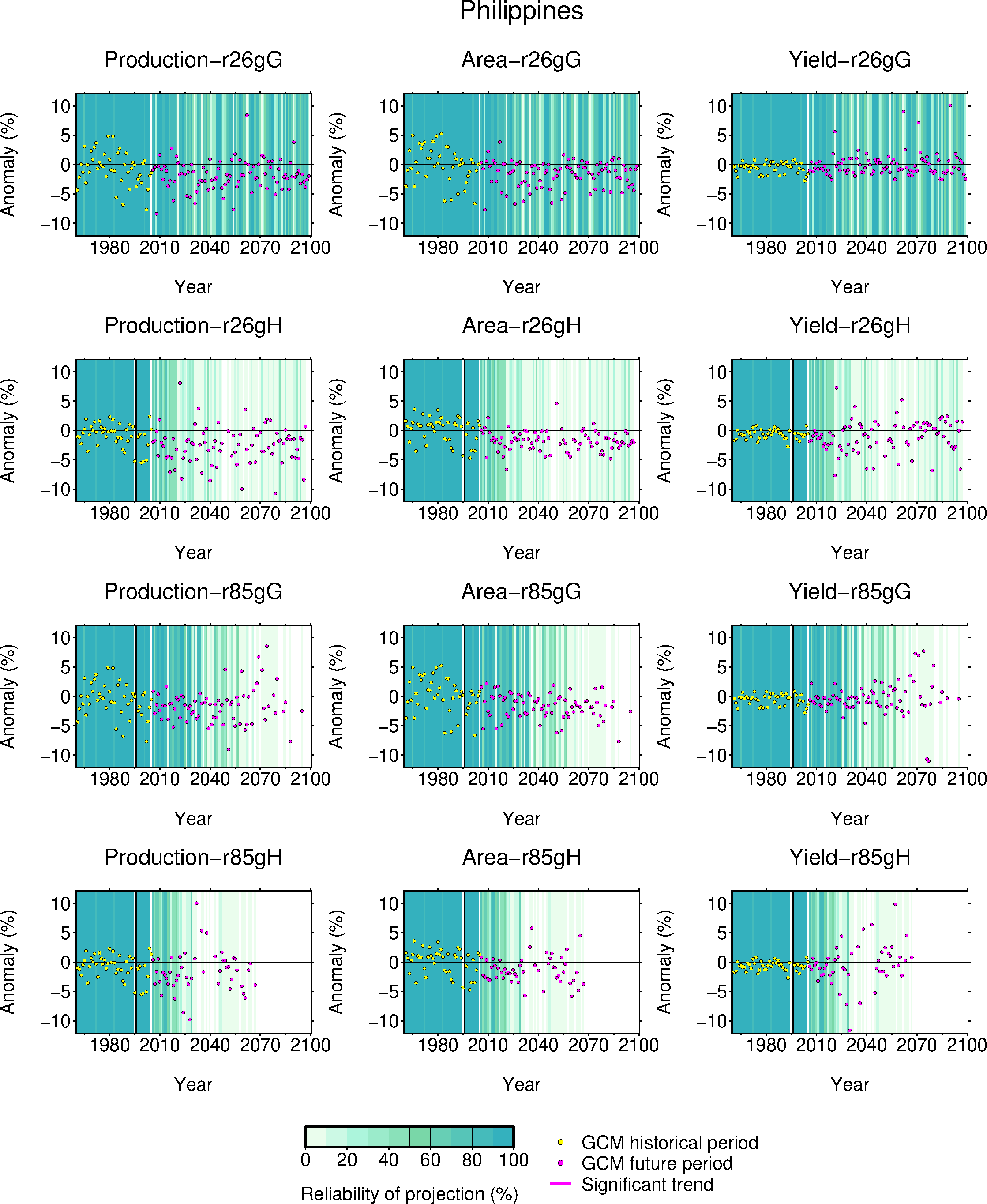


Supplementary Figure 6. (continued)


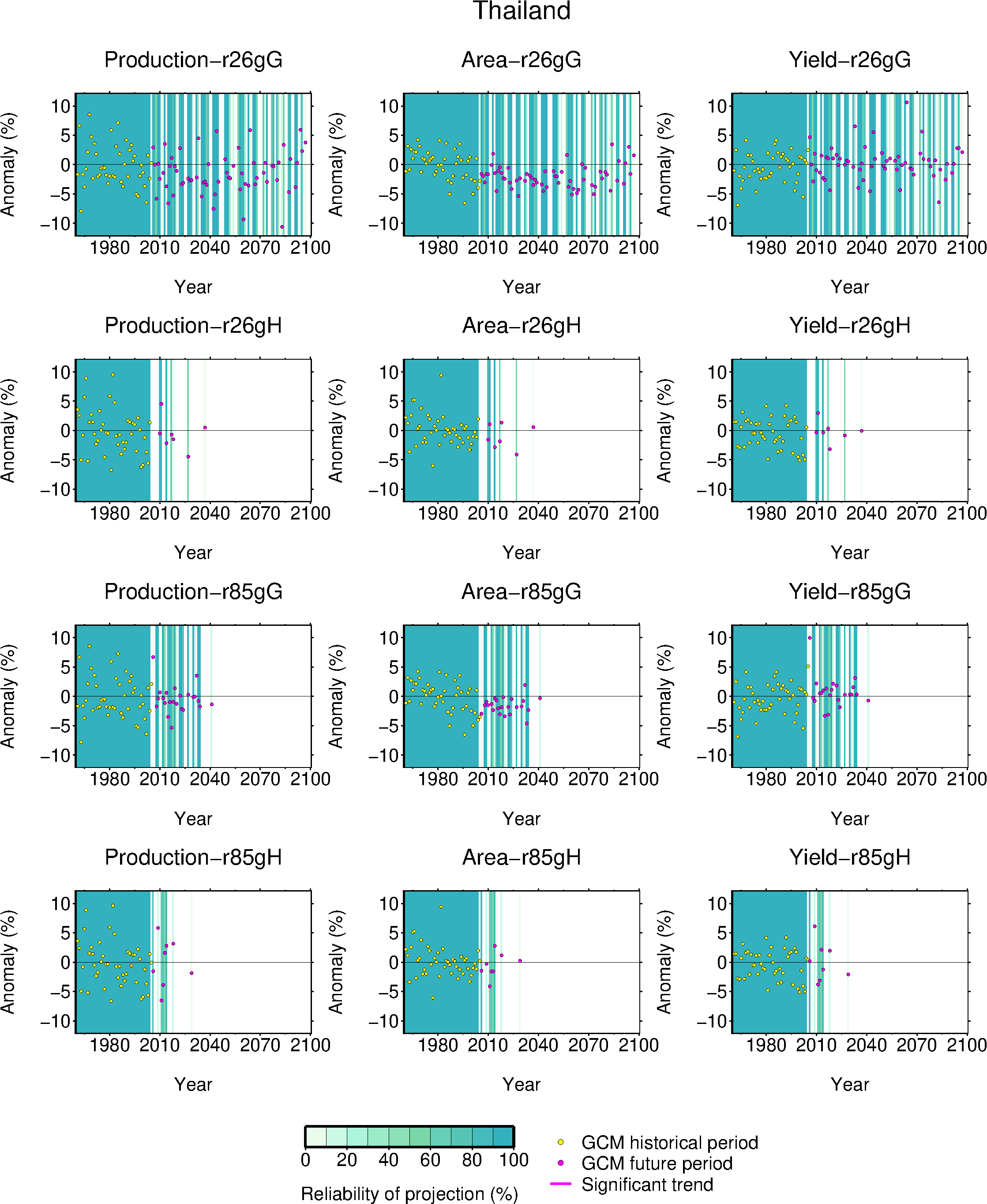


Supplementary Figure 6. (continued)

Supplementary Figure 7. Heatmaps showing the sign of projected changes in production, area, and yield for the period 2006–2100 by climate scenario. +, significant increase; –, significant decrease; and 0, non-significant change at the 5% level. The hatched cells under production indicate whether either, or both, area and yield exhibit significant changes.


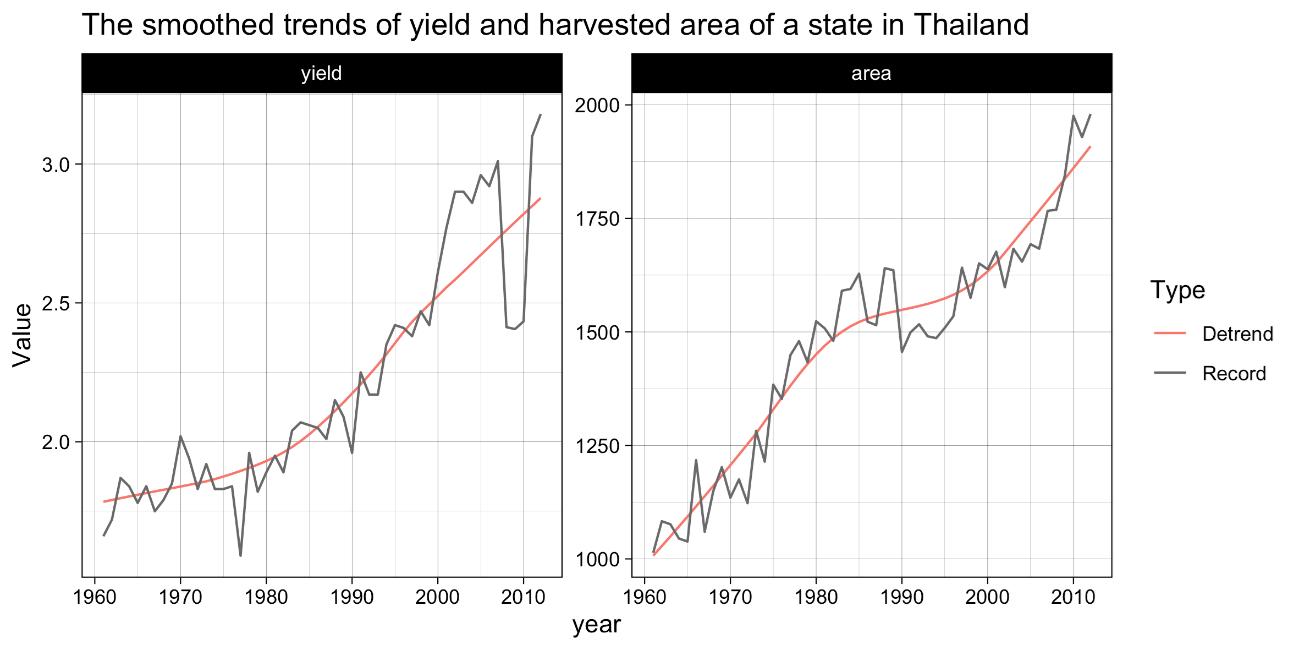


Supplementary Figure 8. Census data of yield (t ha^–1^) and area (10^4^ ha) and their trends. Representative data for Thailand are presented for explanatory purposes. Black, original census data; red, trend estimated using the double-filter approach.


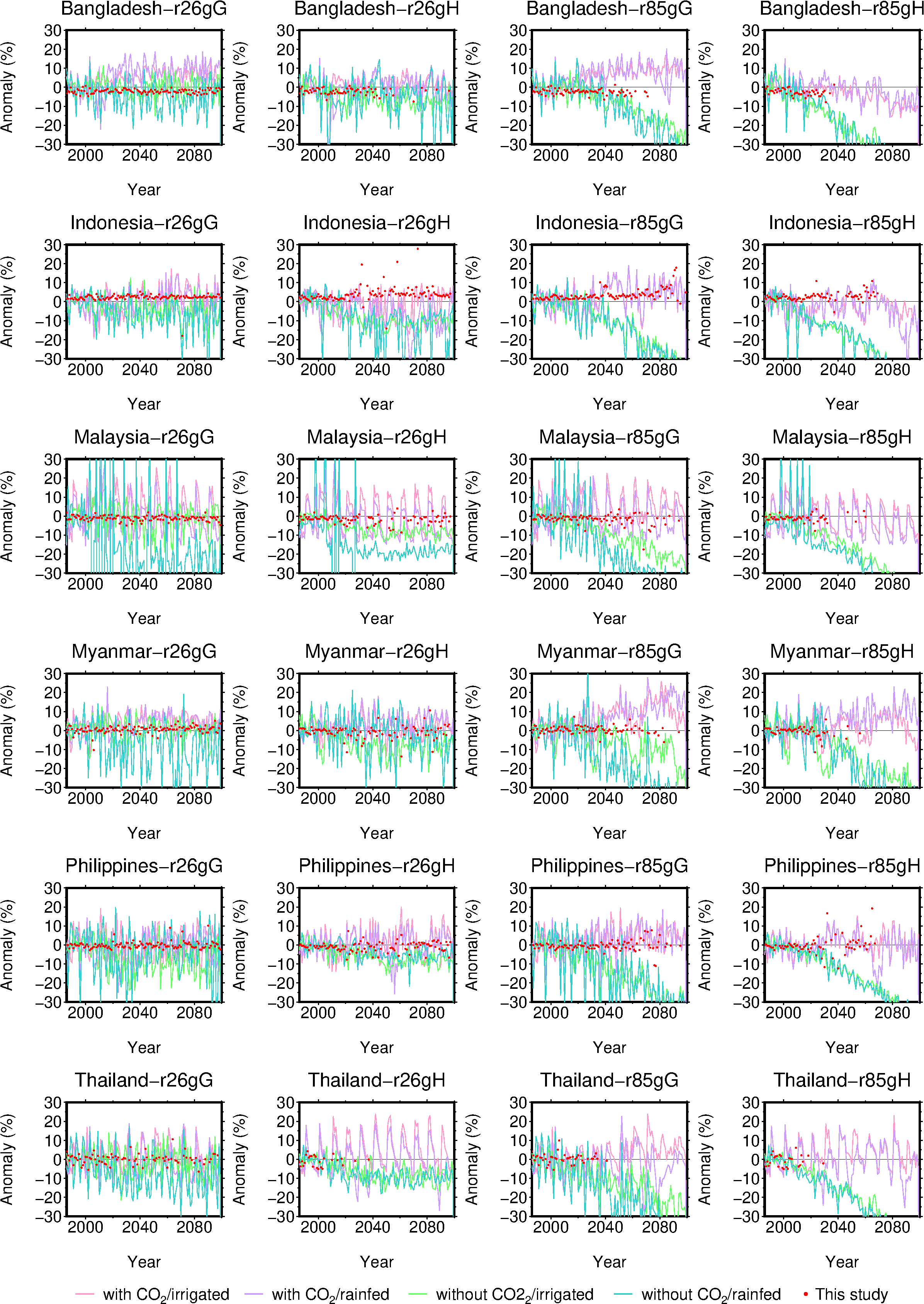


Supplementary Figure 9. Comparisons between two different sources of rice yield projections. One is derived from the elastic net regression models in this study and the other is obtained from AgMIP GGCMs.
